# Umbilical cord management in newborn resuscitation: a systematic review and meta-analysis

**DOI:** 10.1038/s41390-024-03496-7

**Published:** 2024-09-02

**Authors:** Gréta Sz. Major, Vivien Unger, Rita Nagy, Márk Hernádfői, Dániel S. Veres, Ádám Zolcsák, Miklós Szabó, Miklós Garami, Péter Hegyi, Péter Varga, Ákos Gasparics

**Affiliations:** 1 Centre for Translational Medicine, Semmelweis University, Budapest, Hungary; 2 Heim Pál National Pediatric Institute, Budapest, Hungary; 3 Csolnoky Ferenc Hospital, Veszprém, Hungary; 4 Bethesda Children’s Hospital, Budapest, Hungary; 5 Department of Biophysics and Radiation Biology, Semmelweis University, Budapest, Hungary; 6 Department of Neonatology, Semmelweis University, Budapest, Hungary; 7 Pediatric Center, Semmelweis University, Budapest, Hungary; 8 Institute for Translational Medicine, University of Pécs, Pécs, Hungary; 9 Institute of Pancreatic Diseases, Semmelweis University, Budapest, Hungary; 10 Department of Obstetrics and Gynecology, Intensive Neonatal Care Unit, Semmelweis University, Budapest, Hungary

## Abstract

**Background:**

Evidence supporting the benefits of delayed cord clamping is increasing; however, there is no clear recommendation on cord management during newborn resuscitation. This study aimed to investigate the effects of resuscitation initiated with an intact umbilical cord, hypothesizing it is a safe stabilization procedure that improves neonatal outcomes.

**Methods:**

Systematic search was conducted in MEDLINE, Embase, CENTRAL, and Web of Science from inception to March 1, 2024. Eligible articles compared neonatal outcomes in newborns receiving initial stabilization steps before and after cord clamping.

**Results:**

Twelve studies met our inclusion criteria, with six RCTs included in the quantitative analysis. No statistically significant differences were found in delivery room parameters, in-hospital mortality, or neonatal outcomes between the examined groups. However, intact cord resuscitation group showed higher SpO_2_ at 5 min after birth compared to cord clamping prior to resuscitation group (MD 6.67%, 95% CI [−1.16%, 14.50%]). There were no significant differences in early complications of prematurity (NEC ≥ stage 2: RR 2.05, 95% CI [0.34, 12.30], IVH: RR 1.25, 95% CI [0.77, 2.00]).

**Conclusion:**

Intact cord management during resuscitation appears to be a safe intervention; its effect on early complications of prematurity remains unclear. Further high-quality RCTs with larger patient numbers are urgently needed.

**Impact:**

- Initiating resuscitation with an intact umbilical cord appears to be a safe intervention for newborns.
- No statistically significant differences were found in delivery room parameters, in-hospital mortality, and neonatal outcomes between the examined groups.
- The utilization of specialized resuscitation trolleys appears to be promising to reduce the risk of intraventricular hemorrhage in preterm infants.
- Further high-quality RCTs with larger sample sizes are urgently needed to refine recommendations.

## Introduction

The first minutes of life and delivery room management of newborns have a fundamental impact on neonatal mortality and morbidity. Establishing interventions and protocols for resuscitation in the delivery room poses a challenge due to the heterogeneous population as term infants differ from preterm infants who were born at the limit of viability. Recommendations and guidelines on stabilization procedures in the delivery room are constantly changing and evolving by integrating new procedures based on recent evidence (e.g., application of sustained inflations and continuous positive airway pressure (CPAP)).^1–3^

Over the past decades, a number of research studies have examined the physiology and outcomes of delayed cord clamping (DCC); thereby, its numerous beneficial effects on infants have been proven, e.g., improved transitional circulation and iron stores, increased hemoglobin level at birth, decreased need for blood transfusion and lower incidence of intraventricular hemorrhage (IVH) and necrotizing enterocolitis (NEC).^4–6^

Therefore, on the basis of the guidelines influenced by the International Liaison Committee on Resuscitation (ILCOR) recommendations, it is suggested to delay cord clamping (CC) by at least 60 s, ideally after ensuring adequate lung aeration.^7^ However, the timing of CC and the steps of resuscitation are still not synchronized. Unfortunately, non-vigorous and non-breathing infants needing immediate interventions for stabilization are usually clamped immediately and excluded from most studies on different umbilical cord management. In consequence, for preterm and term infants who require resuscitation after birth, we still have insufficient evidence on the optimal time of CC.^4,8,9^ The purpose of our study was to collect all available data on this most vulnerable newborn population in order to discover the effect of intact cords when initiating resuscitation.

## Methods

Our systematic review and meta-analysis is reported based on the recommendations of PRISMA 2020 guideline^10^ (Supplementary Table 1), and as methodological guidance, the Cochrane Handbook^11^ was followed. The prestudy protocol was registered in advance on the International Prospective Register of Systematic Reviews with registration number CRD42022370338. A deviation from the protocol occurred as we also conducted subgroup analyses based on the usage of special resuscitation trolleys. Ethical approval was not required due to the inherent design of the systematic review.

### Eligibility criteria

To address our research question, we included randomized clinical trials (RCTs) and observational studies comparing the initiation of neonatal resuscitation (airway opening maneuvers, positive pressure ventilation, chest compression, etc. except drying and stimulation only) before (intact cord resuscitation (ICR)) and after CC. Conference abstracts and case reports were excluded. Exclusion criteria for the examined population were monochorionic twins, triplets or higher-order multiple pregnancies, major congenital malformations, fetal hydrops, twin-to-twin transfusion syndrome, placental abruption, and placenta previa.

We defined our primary outcomes in advance as in-hospital mortality; presence of IVH (all grades and severe (≥grade 3)), periventricular leukomalacia, and cerebral palsy. Our secondary outcomes included delivery room parameters, early complications of prematurity and maternal outcomes, etc. (see Supplementary Material, sections 2 and 3 and Supplementary Table 2).

### Search strategy and selection process

Our systematic search was conducted in four main databases: MEDLINE (via PubMed), Embase, CENTRAL (the Cochrane Central Register of Controlled Trials), and Web of Science on March 1, 2024, using a predefined search key (see in Supplementary Material, section 4). During the search, no filters or language restrictions were applied. Reference and citation lists of the included studies were examined for further eligible articles using the citationchaser.^12^ After duplicates were removed both automatically and manually, two independent review authors (G.Sz.M. and V.U.) performed the selection process separately via Rayyan (Rayyan Systems, Cambridge, Massachusetts, USA; Qatar Computing Research Institute, Doha, Qatar)^13^ and Endnote 20 (Clarivate Analytics, Philadelphia, Pennsylvania, USA)^14^ reference manager programs. Publications were screened according to the eligibility criteria by title and abstract first and then by full text. Disagreements were resolved by involving the corresponding author (Á.G.).

Data were collected from the eligible articles by two authors (G.Sz.M. and V.U.) independently, using a standardized data collection sheet which was created based on the consensus of clinical and methodological experts. The following data were extracted: title, first author, year of publication, countries, number of centers, study period, DOI (digital object identifier), study design, study population, patient demographics, inclusion and exclusion criteria, interventions, and outcomes measured.

### Study risk of bias (RoB) assessment

The RoB assessment was performed by two authors (G.Sz.M. and V.U.) separately based on the recommendations of the Cochrane Collaboration, using the Cochrane risk-of-bias tool for randomized trials (RoB2) (Cochrane Bias Methods Group, Cochrane Collaboration).^15^ The corresponding author (Á.G.) resolved any occurring disagreements.

### Synthesis methods

As we assumed considerable between-study heterogeneity in all cases, a random-effects model was used to pool effect sizes.

For binary outcomes, risk ratios (RRs) with a 95% confidence interval (CI) were used for the effect size measure. To calculate the study RRs and the pooled RRs, the total number of patients and those with an event of interest in each group were separately extracted from the studies. We reported the results as the risk of an event of interest in the experimental group versus the risk of an event of interest in the control group. For continuous outcomes, differences between the means (MD) with 95% CI were used for effect size measure. To calculate the study MDs and pooled MDs, the sample size, the mean, and the corresponding standard deviation (SD) were extracted from each study. For the Apgar score, the quartiles were reported in most cases (instead of mean and SD). Therefore, differences between group medians (MedD) were used as effect size measures with 95% CI as recommended by McGrath et al. ^16^ We reported the results as an experimental group minus control group values.

Results were considered statistically significant if the pooled CI did not contain the null value. We summarized the findings of the meta-analysis in forest plots. As the study number was small, we did not report the prediction intervals (i.e., the expected range of effects of future studies) of results. Between-study heterogeneity was also described by Higgins&Thompson’s I^2^ statistics.^17^

Subgroup analysis was performed based on gestational age (GA), usage of special resuscitation trolleys, and the type of intervention in the control group.

Small-study publication bias was assessed by visual inspection of funnel plots and calculating Harbord (modified Egger’s) test *p*-value^18^ for RR effect size and classical Egger’s test *p*-value^19^ for MD effect size. Unfortunately, the number of studies was too low; therefore, these assessments were meaningless and were not reported.

Potential outlier publications were planned to be explored using different influence measures and plots following the recommendations of Harrer et al. ^20^. However, the study number was limited; therefore, it was ineffectual to conclude and report.

All statistical analyses were calculated by R software (v4.3.0; R Development Core Team)^21^ using the meta (v6.5.0)^22^ package for basic meta-analysis calculations and plots, and dmetar (v0.0.9000)^23^ package for additional influential analysis calculations and plots.

For additional details see the Supplementary Material, section 5.

### Assessing the level of evidence

To evaluate the quality of evidence, we followed the recommendations of the “Grades of Recommendation, Assessment, Development, and Evaluation (GRADE)” workgroup.^24^

## Results

### Systematic search and selection

The systematic search yielded 17,141 articles. Following duplicate removal and selection processes, we identified 11 eligible studies and an additional one by reference and citation search (Supplementary Fig. 1). In a subset of the qualified papers,^25–29^ the study population included infants for whom it was either uncertain or unnecessary to administer any form of resuscitation following birth. After contacting the corresponding authors for more detailed data, we included one more study^29^ in the analysis. In one article,^30^ we successfully obtained the necessary information needed to determine the total sample size for in-hospital mortality. In conclusion, a total of six RCTs^29–34^ were included in our quantitative analysis, and ten RCTs^25–34^, and two observational studies^35,36^ in the systematic review. Details and results of the studies included in the systematic review are summarized in Supplementary Table 3.

### Study characteristics

The baseline characteristics of studies included in the meta-analysis can be found in Table 1, and the detailed interventions of the examined groups and the respiratory status at the time of CC are summarized in Table 2. We analyzed six RCTs including 610 preterm^29,31–34^ and term^30,34^ infants. The timing of CC and the rates of different resuscitation procedures were heterogeneous among the studies. In the intervention group, newborns received DCC at various time points (either at 50 s,^32^ at 60 s,^29,31^ at least 180 s^30,34^) or physiological-based cord clamping (PBCC).^33^ In the control group, immediate/early cord clamping (ICC/ECC) (<60 s)^29,30,34^ or DCC (>30–60 s)^31–33^ were performed. Therefore, besides the GA, we also performed subgroup analyses based on the type of intervention in the control group and the usage of a special resuscitation trolley (see in the Supplementary Material, Figs. 16–31)

**Table 1:** Baseline characteristics of studies included in the meta-analysis.

First author and year of publication	Country	Study design	Inclusion criteria	Exclusion criteria	Number of patients, (*n*)	Gestational age, (weeks)	Birthweight, (g)	Females, (%)	Measured outcomes (and included in the analysis)
Andersson et al.^30^	Nepal	RCT	Infants born at GA ≥ 33 weeks, in need of resuscitation: no breathing despite thorough drying and additional stimulation within 30 s after birth, uncomplicated pregnancies, no complication at hospital admission, healthy mothers (no clinical history of hypertension, infection, diabetes, or chronic medical condition), expected vaginal delivery and singleton pregnancy	Monochorionic twins (from an ultrasound scan) or clinical evidence of twin–twin transfusion syndrome, triplets or higher order multiple pregnancies, fetuses with known congenital malformation	I: 74 C: 48	I: NA C: NA	I: NA C: NA	NA	In-hospital mortality, SpO_2_ at 5 min and 10 min after birth, Apgar score at 1 min after birth
Finn et al.^29^	Ireland	RCT	Preterm infants born at GA < 32 weeks	Major congenital anomaly, bleeding from placenta previa, placental abruption or accreta, twin-to-twin transfusion syndrome, hydrops, and cord prolapse	I: 14 C: 12	I: 28 [26.4–29.6] C: 28.5 [25.7–30.5]	I: 925 [630–1490] C: 1080 [755–1613]	NA	Severe IVH, NEC ≥ grade 2, ROP requiring treatment, BPD, need for transfusion, need for phototherapy, need for surfactant therapy, LOS, Apgar score at 1 min after birth, temperature at admission to the NICU
Katheria et al.^31^	USA	RCT	Preterm infants born at GA < 32 weeks	Monochorionic multiples, placenta previa, concern for an actual abruption, Rh sensitization, hydrops, and congenital anomalies	I: 75 C: 75	I: CS: 28.25 ± 2.41, V: 29 ± 3 C: CS: 28.47 ± 2.17, V: 28 ± 3	I: CS: 1184.94 ± 355.72, V: 1435 ± 424 C: CS: 1174.19 ± 407.037, V: 1260 ± 419	I: CS: 44.45, V: 17 C: CS: 62.91, V: 31	In-hospital mortality, all grades, and severe IVH, NEC ≥ grade 2, ROP requiring treatment, BPD, PDA requiring treatment, need for transfusion, need for surfactant therapy, Apgar score at 1 min and 5 min after birth, the temperature at admission to the NICU
Knol et al.^33^	The Netherlands	RCT	Infants born vaginally or by cesarean section <32 weeks of GA	Significant congenital malformations influencing cardiopulmonary transition, placental abruption, placenta praevia, and signs of severe fetal distress necessitating emergency cesarean section	I: 20 C: 17	I: 28^+4^ [27^+6^–30^+3^] C: 30^+2^ [27^+5^–31^+0^]	I: 1155 [1043–1349] C: 1200 [895–1620]	I: 80 C: 47.1	In-hospital mortality, all grades, and severe IVH, NEC ≥ grade 2, BPD, PDA requiring treatment, need for transfusion, need for phototherapy, need for surfactant therapy, LOS, Apgar score at 1 min and 5 min after birth, the temperature at admission to the NICU
Nevill et al.^32^	New Zealand	RCT	Preterm infants less than 31 weeks of gestation undergoing DCC, provided they were either not breathing or making irregular nonsustained breathing efforts during DCC	Severe fetal growth restriction, twin-to-twin transfusion syndrome, maternal compromise, placental abruption, and known severe congenital malformation, infants who established regular breathing movements or were crying by 15 s, infants who were apneic and also flaccid and pale	I: 57 C: 56	I: 28 [25–29] C: 27 [26–29]	I: 1100 [817–1290] C: 1044 [818–1360]	I: 51 C: 47	In-hospital mortality, all grades, and severe IVH, NEC ≥ grade 2, ROP requiring treatment, BPD, PDA requiring treatment, need for transfusion, need for phototherapy, need for surfactant therapy, LOS, SpO_2_ at 5 min and 10 min after birth, Apgar score at 1 min and 5 min after birth
Raina et al.^34^	India	RCT	Neonates born at ≥34 weeks of gestation to women with pregnancy or labor complications and requiring resuscitation at birth	Congenital malformation or chromosomal the fetus, fetal hydrops, monochorionic or monoamniotic placentation, triplets, and higher-order pregnancy, abruption placenta or cord abnormalities, placenta accreta or percreta, anterior placenta previa (in case of cesarean delivery), or ruptured uterus	I: 71 C: 91	I: 37 ± 1.8 C: 37.2 ± 2	I: 2442 ± 643 C: 2488 ± 639	I: 45.1 C: 56.1	In-hospital mortality, need for phototherapy, SpO_2_ at 5 min and 10 min after birth, Apgar score at 1 min and 5 min after birth

mean ± SD; median [IQR].

*SD* standard deviation, *IQR* interquartile range, *RCT* randomized controlled trial, *GA* gestational age, *s* second(s), *I* intervention group, *C* control group, *NA* not available, *SpO*_*2*_ oxygen saturation level, *IVH* intraventricular hemorrhage, *NEC* necrotizing enterocolitis, *ROP* retinopathy of the prematurity, *BPD* bronchopulmonary dysplasia, *LOS* late-onset sepsis, *NICU* neonatal intensive care unit, *USA* United States of America, *CS* cesarean section, *V* vaginal delivery, *PDA* patent ductus arteriosus, *DCC* delayed cord clamping.

**Table 2:** Detailed interventions and respiratory status of the infants at the time of CC in the examined groups.

First author and year of publication	Timing of CC				Required resuscitation				Respiratory status	
	Timing of CC in the intervention group: planned; actual, (s)	Timing of CC in the control group: planned; actual, (s)	Deviation from the planned timing of CC in the intervention group, (%)	Deviation from the planned timing of CC in the control group, (%)	Type of required resuscitation in the intervention group	Type of required resuscitation in the control group	Deviation from the allocated intervention in the intervention group, (%)	Deviation from the allocated intervention in the control group, (%)	Spontaneous breathing babies before CC in the intervention group, (%)	Spontaneous breathing babies before CC in the control group, (%)
Andersson et al.^30^	DCC > 180; 187 [42–195]	ECC < 60; 25 [11–40]	NA	None	Bag and mask ventilation	Bag and mask ventilation	None	None	NA time to regular breathing (s): 78 [67–155]	NA time to regular breathing (s): 356 [98–389]
Finn et al.^29^	DCC at 60	ICC < 20	None	None	Vast majority received CPAP (or more)	Vast majority received CPAP (or more)	14% Did not receive ICR	None	Spontaneous respirations at 60 s: 85,7%	Spontaneous respirations at 60 s: 66.7%
Katheria et al.^31^	DCC at 60; appr. 65	DCC at 60; appr. 65	5% Received ECC	5% Received ECC	CPAP only (44%), PPV (59%), intubation (36%)	CPAP only (NA), PPV (69%), intubation (44%)	NA	NA	CS: 92%, vaginal delivery: 100%, all: 93%	CS: 90%, vaginal delivery: 84.61%, all: 89%
Knol et al.^33^	PBCC (CC when HR > 100 bpm and SpO_2_ > 90% while using FiO_2_ < 40%); 349 ± 157	DCC at 30–60; 62 ± 30	None	None	CPAP (90%), PPV (70%), intubation (5%)	CPAP (100%), PPV (58.8%), intubation (0%)	10% Did not receive ICR	None	NA time to stabilization (regular spontaneous breathing, HR ≥ 100 bpm, SpO_2_ > 90% while FiO_2_ < 0,4) (s): ITT: 354 ± 147, as treated: 325 ± 95	NA time to stabilization (regular spontaneous breathing, HR ≥ 100, SpO_2_ > 90 while FiO_2_ < 0,4) (s): ITT: 427 ± 174, as treated: 445 ± 190
Nevill et al.^32^	DCC at 50	DCC at 50	5% Received ECC	9% Received ECC	CPAP (25%), IPPV (60%,) intubation (16%), chest compression (5%), adrenalin (0%)	CPAP (30%), IPPV (45%), intubation (21%), chest compression (12.5%), adrenalin (3.6%)	1 Case (1.8%) wrong intervention received		No increase in the number of spontaneously breathing infants in the intervention vs. control group at 60 s	
Raina et al.^34^	DCC at least 180 s or when the neonate exhibited spontaneous breathing whichever was later with a maximum of 5 min; 180 [180–180]	ECC < 30; 20 [15–20]	24% Received CC < 180 s	None	PPV (100%), intubation (7%), chest compression (0%), adrenalin (0%)	PPV (100%), intubation (8.8%), chest compression (2.2%), adrenalin (1.1%)	None	None	All	None

mean ± SD; median [IQR].

*CC* cord clamping, *s* second(s), *min* minute(s), *DCC* delayed cord clamping, *ECC* early cord clamping, *ICC* immediate cord clamping, *PBCC* physiological-based cord clamping, *CPAP* continuous positive airway pressure, *PPV* positive pressure ventilation, *IPPV* intermittent positive-pressure ventilation, *ICR* intact cord resuscitation, *CS* cesarean section, *HR* heart rate, *bpm* beat per minute, *SpO*_*2*_ oxygen saturation level, *FiO*_*2*_ fraction of inspired oxygen, *ITT* intention-to-treat analysis, *NA* not available.

### In-hospital mortality

**Fig. 1:**
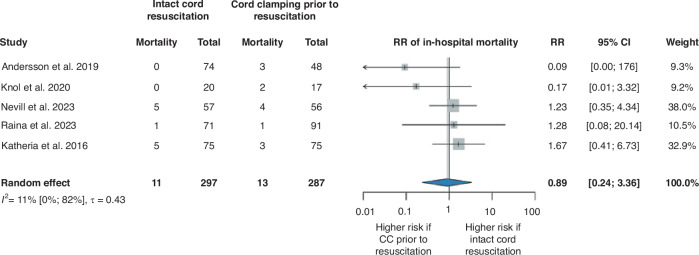
Forest plot representing the risk ratio of in-hospital mortality in infants who received ICR or CC prior to resuscitation after birth. RR risk ratio, 95% CI 95% confidence interval, CC cord clamping.

Analysis of in-hospital mortality included five RCTs^30–34^ and involved 584 patients (Fig. 1). This event occurred in 11 out of 297 patients assigned to the ICR group and 13 out of 287 patients assigned to the CC prior to resuscitation group, which indicates no significant difference between the examined groups (RR 0.89, 95% CI [0.24, 3.36]).

No significant differences were found between the groups either in our subgroup analyses (Supplementary Figs. 2–4), or in any studies included in our systematic review and examined this outcome^25,28,35,36^ (Supplementary Table 3).

### Delivery room parameters (oxygen saturation level, Apgar score) and temperature at NICU admission

**Fig. 2:**
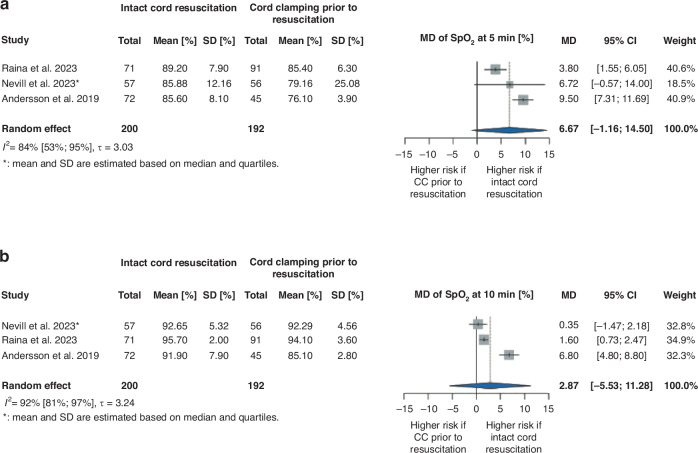
Forest plot representing the mean difference of SpO_2_ after birth in infants who received ICR or CC prior to resuscitation after birth. **a** Forest plot representing the mean difference of SpO_2_ at 5 min after birth in infants who received ICR or CC prior to resuscitation after birth. **b** Forest plot representing the mean difference of SpO_2_ at 10 min after birth in infants who received ICR or CC prior to resuscitation after birth. SpO_2_ oxygen saturation level by pulse oximetry, min minutes, MD mean difference, SD standard deviation, 95% CI 95% confidence interval, CC cord clamping.

The pooled analysis of three RCTs^30,32,34^ with 392 patients showed higher mean SpO_2_ at 5 min (MD 6.67%, 95% CI [−1.16%, 14.50%]) and 10 min (MD 2.87%, 95% CI [−5.53%, 11.28%]) after birth in the ICR group; however, the difference was not statistically significant (Fig. 2a, b).

**Fig. 3:**
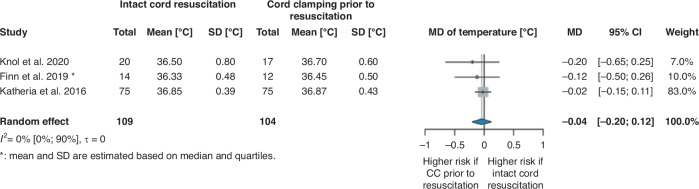
Forest plot representing the mean difference of temperature at admission to the NICU in infants who received ICR or CC prior to resuscitation after birth. NICU neonatal intensive care unit, MD mean difference, SD standard deviation, 95% CI 95% confidence interval, CC cord clamping.

We found no significant difference between the examined groups in terms of Apgar score at 1 min and 5 min after birth (1 min MedD –0.09, 95% CI [−0.55, 0.36]^29,31–34^ and 5 min MedD –0.03, 95% CI [−0.36, 0.29]^31–34^) (Supplementary Figs. 5 and 6) and temperature at admission to the NICU (MD −0.04 °C, 95% CI [−0.20 °C, 0.12 °C]) (Fig. 3).

In the articles included in our systematic review that examined Apgar scores,^26–28,35,36^ SpO_2_ shortly after birth^27,28^ and temperature at NICU admission,^25,28,35^ no significant difference was found between the intervention and control groups (Supplementary Table 3).

In terms of heart rate (HR), Badurdeen et al. ^26^ observed that PBCC resulted in a similar mean HR between 60 s to 120 s after birth compared to infants receiving ECC. Andersson et al. ^30^ reported significantly lower HR values in the ICR group than in the ECC group at 1 min and 5 min after birth. Raina et al. ^34^ also found significantly lower HRs in the ICR group than in the ECC resuscitation group at 5 and 10 min. In the study of Hoeller et al. ^36^, the PBCC group had lower HRs during the first 72 h of life than those who underwent standard DCC, reaching significance by 10 h of monitoring (Supplementary Table 3).

### Early complications of prematurity (IVH, NEC, ROP, BPD, PDA, and LOS)

**Fig. 4:**
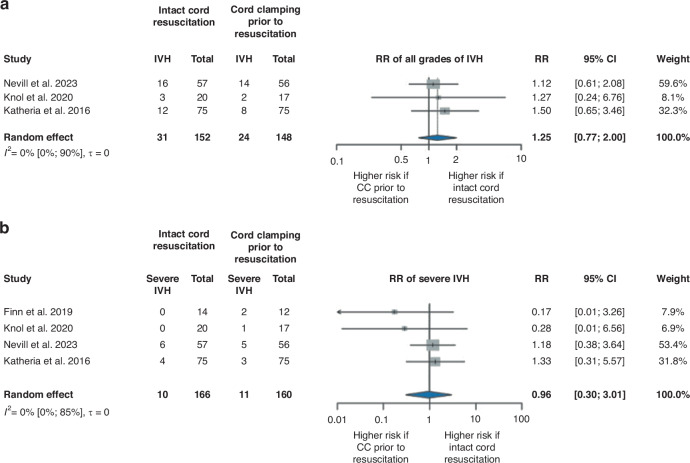
Forest plot representing the risk ratio of IVH in infants who received ICR or CC prior to resuscitation after birth. **a** Forest plot representing the risk ratio of all grades of IVH in infants who received ICR or CC prior to resuscitation after birth. **b** Forest plot representing the risk ratio of severe IVH (≥grade 3) in infants who received ICR or CC prior to resuscitation after birth. IVH intraventricular hemorrhage, RR risk ratio, 95% CI 95% confidence interval, CC cord clamping.

For the analysis of all grades of IVH, we had three articles^31–33^ involving 300 patients, and 55 infants with IVH (Fig. 4a). The overall effect size was RR of 1.25, 95% CI [0.77, 2.00]. Regarding severe ( ≥ grade 3) IVH, we had four studies^29,31–33^ covering 326 patients with an event number of 21 patients (Fig. 4b). The rate of severe IVH showed no significant difference between the groups (RR 0.96, 95% CI [0.30, 3.01]), although we found a RR of 0.75, 95% CI [0.06, 10.11] when we examined only those studies that used specialized resuscitation trolleys (Supplementary Fig. 7).

No significant differences were found between the intervention and control groups in articles included in our systematic review that examined all grades^25,36^ and severe^25,28^ IVH, with the exception of the article by Hocq et al. ^35^ who reported that the incidence of all grades of IVH decreased during the ICR implementation period (Supplementary Table 3).

**Fig. 5:**
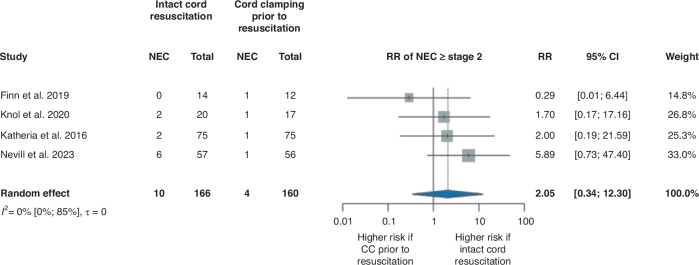
Forest plot representing the risk ratio of NEC ≥ stage 2 in infants who received ICR or CC prior to resuscitation after birth. NEC necrotizing enterocolitis, RR risk ratio, 95% CI 95% confidence interval, CC cord clamping.

The analysis of four studies^29,31–33^ involving 326 patients and 14 outcome events resulted in an RR of 2.05, 95% CI [0.34, 12.30] for NEC ≥ stage 2, although it did not reach significance level (Fig. 5). When we analyzed only studies that compared ICR to DCC prior to resuscitation,^31–33^ the RR for NEC ≥ stage 2 was 2.89, 95% CI [0.51, 16.40]; however, this result was not statistically significant (Supplementary Fig. 8). Analyzing studies^29,31,33^ where newborn stabilization was performed with a special resuscitation trolley, we found an RR of 1.22, 95% CI [0.12, 12.85] but it did not reach a significance level either (Supplementary Fig. 9).

Hocq et al. ^35^ reported a lower incidence of NEC ≥ stage 2 during the ICR implementation period. Further studies examining this outcome^25,28,36^ did not find any significant differences between the examined groups (Supplementary Table 3).

**Fig. 6:**
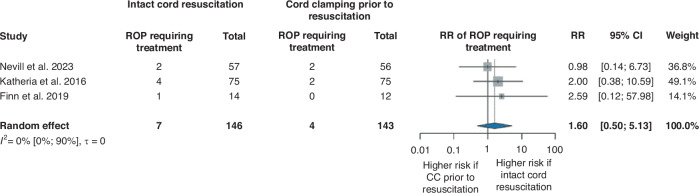
Forest plot representing the risk ratio of ROP requiring treatment in infants who received ICR or CC prior to resuscitation after birth. ROP retinopathy of the prematurity, RR risk ratio, 95% CI 95% confidence interval, CC cord clamping.

ROP requiring treatment analysis resulted in a RR of 1.60, 95% CI [0.50, 5.13]^29,31,32^ (Fig. 6), and for BPD, we found a RR of 1.20, 95% CI [0.83, 1.76]^29,31–33^ (Supplementary Fig. 10), these findings did not reach significance level. In PDA requiring treatment (RR 0.88, 95% CI [0.47, 1.66])^31–33^ (Supplementary Fig. 11) and LOS (RR 0.91, 95% CI [0.44, 1.87])^29,32,33^ (Supplementary Fig. 12), there were no clinically or statistically significant differences between the two groups.

No significant differences were found between the intervention and control groups in articles included in our systematic review that examined ROP,^25,28,35,36^ BPD,^25,35,36^ PDA^25,35^ and LOS^28,36^ (Supplementary Table 3).

### Need for blood transfusion and surfactant therapy in NICU

We found no significant differences between the groups in the need for blood transfusion (RR 0.95, 95% CI [0.73, 1.25])^29,31–33^ (Supplementary Fig. 13) and need for surfactant therapy (RR 0.96, 95% CI [0.75, 1.22])^29,31–33^ (Supplementary Fig. 14).

No significant differences were observed between the groups in any of the articles included in our systematic review that examined the need for blood transfusion^25,35^ and surfactant therapy^35^ (Supplementary Table 3).

### Safety parameters

In our study, we included the outcomes of the need for phototherapy, hypothermia (<36.0 °C) at NICU admission, and maternal outcomes including maternal blood loss, pp. hemorrhage, and pp. infection as safety parameters.

We found no significant differences between the intervention and control groups in the need for phototherapy (RR 1.10, 95% CI [0.92, 1.30])^29,32–34^ (Supplementary Fig. 15) and studies^25,26,35^ included in our systematic review that examined this outcome found no differences either (Supplementary Table 3).

For hypothermia^25,28,33,35^ and maternal outcomes (blood loss,^26,32,33^ pp. hemorrhage,^25,26,28,32,33^ pp. infection^25,26,28,32^) no significant differences were found between the examined groups (Supplementary Table 3).

### RoB assessment and quality of evidence

The RoB assessment is summarized in Supplementary Tables 4 and 5. Four trials^29–31,33^ had a high RoB due to deviations from the intended interventions. The level of evidence is presented in Supplementary Table 6, for most of our outcomes the GRADE assessment resulted in low certainty due to serious RoB and imprecision, but certainty had to be downgraded to very low due to serious RoB and very serious imprecision in some cases. The assessment of small study bias was meaningless as we had only a few studies.

## Discussion

The present systematic review and meta-analysis examined the optimal umbilical cord management during neonatal resuscitation. According to the resuscitation guidelines influenced by the ILCOR recommendations, delaying CC by a minimum of 60 s is recommended, preferably following adequate lung aeration.^7^ A recent systematic review and network meta-analysis with individual participant data on preterm infants found that the highest reduction in mortality occurred when CC was deferred for at least 120 s. Furthermore, the study suggests that resuscitation with an intact cord might be beneficial, but more evidence is needed to support this practice.^37^ The major limitations of the first quantitative analysis examining this question conducted by Avinash et al. ^38^ were the limited number of studies available for inclusion and their relatively small sample sizes.

Currently, there are well-defined protocols for cases where CC precedes newborn resuscitation. In cases where initial resuscitation interventions and appropriate thermal care can be safely performed with an intact cord without compromising the newborn, CC may be delayed during these interventions.^7,8^ Therefore, it is essential to establish standardized procedures and equipment for ICR. However, this concept lacks robust evidence; consequently, explicit protocols and equipment for ICR have not yet been defined.

### Main findings

In our study, no statistically significant differences were found in terms of in-hospital mortality, delivery room parameters, and early complications of prematurity. Intact cord management during resuscitation appears to be safe and may improve initial oxygenation, although this is in conflict with current standards for delivery room resuscitation.

In some of the examined outcomes, we observed the following findings which might be relevant in patient care.

#### Delivery room parameters

Although our results did not reach the statistically significant level, they suggest a possible beneficial effect of ICR on oxygenation. This finding was also noted in another study^27^ that was excluded from our analysis because of the population examined. In addition to CC timing, FiO_2_ is another critical factor in neonatal stabilization. Current international guidelines recommend starting resuscitation with 21–30% FiO_2_ to mitigate the potential for hyperoxia-induced tissue damage.^39^ All included studies reporting on this aspect^30–33^ used an initial FiO_2_ < 40%. Nevertheless, findings from both animal and human studies indicate the potential benefits of starting resuscitation with 100% FiO_2_.^40–42^ As further larger investigations are necessary for conclusive evidence, the ongoing DOXIE trial was conducted to directly compare the use of 30% FiO_2_ to 100% FiO_2_ during ICR.^43^

Due to a lack of data, we could not perform an HR analysis, which is an important delivery room parameter; however, studies reporting data on this aspect^26,30,34,36^ found lower HR values after birth in the ICR group which is hypothesized to be a result of the increased blood volume following ICR.^36^

#### Early complications in preterm infants

However, the results of early preterm complications did not show statistically significant differences between the groups, interestingly, the risk of NEC ≥ stage 2 and treatment-requiring ROP seemed to be higher in the ICR group. Examining studies that were excluded from the analysis because of the population or the study design, Deng et al. ^28^ also reported higher rates of NEC ≥ stage 2 and ROP ≥ phase 2 in the DCC + nCPAP group compared to the DCC-only group, but these results did not reach statistical significance either. In contrast, Hocq et al. ^35^ found a lower incidence of NEC ≥ stage 2 following ICR implementation in their hospital protocol. Free radicals and hyperoxia might play a role in the development of NEC and ROP.^44–46^

#### Resuscitation trolleys

To provide continuous placenta-newborn connection, while allowing an immediate stabilization of non-vigorous newborns, different resuscitation platforms were developed such as LifeStart Trolley (Inspiration HealthCare Group PLC, Croydon, UK), Concord Birth Trolley (Leiden University Medical Center, Leiden, Netherlands), NOOMA cart (Maternal Life, LLC, Palo Alto, California, USA) and INSPiRe Trolley (Integrated Neonatal Support on Placental Circulation with Resuscitation, Alberta Health Services, Edmonton, Alberta, Canada).^47^ Although resuscitation with an intact cord can also be achieved with standard equipment, certain challenges such as the warming device to prevent hypothermia and the availability of sufficient respiratory support may persist.^48^ Among the RCTs we included in our meta-analysis, ICR has performed with^29,31,33,34^ and without^30,32^ the use of specialized resuscitation trolleys as well. A subgroup analysis revealed that employing specialized trolleys might improve the impact of ICR on early complications associated with prematurity: although the rate of severe IVH did not significantly differ between the groups in the analysis of all studies included, when examining specifically those using special equipment, there appeared to be a lower risk of severe IVH in the ICR group compared to the CC prior to the resuscitation group. For NEC ≥ stage 2, although the risk was still higher in the ICR group, it was almost halved when a special trolley was used. However, conducting a subgroup analysis resulted in an even smaller sample size and none of these results reached significance.

#### Common concerns about DCC

For DCC, safety parameters include hypothermia, the necessity for phototherapy, maternal blood loss, and pp. infection. In our analysis, we did not observe significant differences in the need for phototherapy. Although we did not have sufficient data to perform a statistical analysis for the rest of the outcomes, studies^25,26,28,32,33^ reporting data on these did not find any significant difference between the groups, except Knol et al. ^33^ and Hocq et al. ^35^ who found moderate hypothermia on admission to NICU in a higher proportion of very preterms receiving ICR than the control group.

### Strengths and limitations

In this systematic review and meta-analysis, we aimed to achieve the highest level of evidence available; therefore, we followed our pre-registered protocol. Studies were included only if they provided explicit information indicating that all or nearly all newborns in both arms had received resuscitation after birth. We conducted a quantitative analysis of the eligible RCTs and included all studies examining this specific question in the systematic review part. As we had a broad population (preterm and term infants) and heterogeneous interventions to examine a wide range of outcomes, we conducted subgroup analyses where feasible to mitigate the effect of these factors.

Due to the inclusion criteria and the early stage of ICR implementation in clinical practice, a limited number of studies were eligible, posing limitations to our analysis. The generalizability of our findings is challenged by the small sample size and number of events (sometimes zero). In addition, protocols of interventions and definitions of outcomes were heterogeneous or even missing in some cases. Devices used for bedside newborn stabilization can be crucial; however, different resuscitation platforms were used in the studies included. Another limitation was the presence of a moderate and high RoB in most domains. Therefore, caution is needed when interpreting our results.

### Implications for clinical practice and future research

The translation of scientific findings into daily practice plays a key role, highlighting that effective implementation significantly improves the quality and cost-efficiency of healthcare. This process is essential to ensure that advancements in medical research directly benefit patient care and public health.^49,50^

The potential beneficial effect of ICR on oxygenation in the population of term infants suggests that recommending ICR may be justified. Nevertheless, ICR should be applied in preterm infants with caution, and we recommend that this practice be performed only in specialized centers with appropriate expertise, protocols, and equipment, given the potential for complications. Based on our results the use of special resuscitation trolleys appears to be beneficial.

There is still no clear and strong evidence for optimal cord management during neonatal resuscitation. There are ongoing multicenter RCTs^51–54^ to examine this question and we encourage researchers to conduct further high-quality RCTs with large sample sizes, homogeneous intervention protocols, and outcome definitions. In addition, it is essential to differentiate outcome data between infants requiring post-birth stabilization measures and those not requiring them. This approach increases the representativeness of the results.

## Conclusion

Intact cord management during resuscitation appears to be safe and may improve initial oxygenation, although this is in conflict with current standards for delivery room resuscitation. The early complications of prematurity remain unclear. The use of specialized resuscitation trolleys seems promising to reduce the risk of IVH. There is an urgent need for further high-quality RCTs with larger patient numbers, especially with specialized resuscitation trolleys and physiological-based CC.

## Supplementary information

‏‏‏‏Supplementary Material

## Data Availability

The datasets used in this study can be found in the full-text articles included in the systematic review and meta-analysis.
