# Supplementary material for: Umbilical cord management in newborn resuscitation: a systematic review and meta-analysis
Source: Pediatr Res. 2024 Sep 2;97(5):1481–91. doi: 10.1038/s41390-024-03496-7 (PMC12119367; doi:10.1038/s41390-024-03496-7)
Supplement: Supplementary file 1 — ‏‏‏‏Supplementary Material [file 41390_2024_3496_MOESM1_ESM.pdf]

SUPPLEMENTARY MATERIAL

Title

Umbilical Cord Management in Newborn Resuscitation: A Systematic Review and Meta-analysis

Authors

Gréta Sz Major[1,2], Vivien Unger[1,3], Rita Nagy[1,2], Márk Hernádfői[1,4], Dániel S Veres[1,5], Ádám Zolcsák[1,5], Miklós Szabó[1,6], Miklós Garami[1,7], Péter Hegyi[1,8,9], Péter Varga[1,6,10], Ákos Gasparics* [1,6,10]

Affiliations

1. Centre for Translational Medicine, Semmelweis University, Budapest, Hungary

2. Heim Pál National Pediatric Institute, Budapest, Hungary

3. Csolnoky Ferenc Hospital, Veszprém, Hungary

4. Bethesda Children's Hospital, Budapest, Hungary

5. Department of Biophysics and Radiation Biology, Semmelweis University, Budapest, Hungary

6. Department of Neonatology, Semmelweis University, Budapest, Hungary

7. Pediatric Center, Semmelweis University, Budapest, Hungary

8. Institute for Translational Medicine, University of Pécs, Pécs, Hungary

9. Institute of Pancreatic Diseases, Semmelweis University, Budapest, Hungary

10. Department of Obstetrics and Gynecology, Intensive Neonatal Care Unit, Semmelweis University, Budapest, Hungary

1. PRISMA 2020 Checklist

Section and Topic	Item #	Checklist item	Location where item is reported
**TITLE**			
Title	1	Identify the report as a systematic review.	p1
**ABSTRACT**			
Abstract	2	See the PRISMA 2020 for Abstracts checklist.	p3
**INTRODUCTION**			
Rationale	3	Describe the rationale for the review in the context of existing knowledge.	p4
Objectives	4	Provide an explicit statement of the objective(s) or question(s) the review addresses.	p4
**METHODS**			
Eligibility criteria	5	Specify the inclusion and exclusion criteria for the review and how studies were grouped for the syntheses.	p5
Information sources	6	Specify all databases, registers, websites, organisations, reference lists and other sources searched or consulted to identify studies. Specify the date when each source was last searched or consulted.	p5-6
Search strategy	7	Present the full search strategies for all databases, registers and websites, including any filters and limits used.	p5-6
Selection process	8	Specify the methods used to decide whether a study met the inclusion criteria of the review, including how many reviewers screened each record and each report retrieved, whether they worked independently, and if applicable, details of automation tools used in the process.	p5-6
Data collection process	9	Specify the methods used to collect data from reports, including how many reviewers collected data from each report, whether they worked independently, any processes for obtaining or confirming data from study investigators, and if applicable, details of automation tools used in the process.	p6

Section and Topic	Item #	Checklist item	Location where item is reported
Data items	10a	List and define all outcomes for which data were sought. Specify whether all results that were compatible with each outcome domain in each study were sought (e.g. for all measures, time points, analyses), and if not, the methods used to decide which results to collect.	p5, Suppl. p8, Suppl. Tables 2-3
	10b	List and define all other variables for which data were sought (e.g. participant and intervention characteristics, funding sources). Describe any assumptions made about any missing or unclear information.	Tables 1 and 2
Study risk of bias assessment	11	Specify the methods used to assess risk of bias in the included studies, including details of the tool(s) used, how many reviewers assessed each study and whether they worked independently, and if applicable, details of automation tools used in the process.	p6
Effect measures	12	Specify for each outcome the effect measure(s) (e.g. risk ratio, mean difference) used in the synthesis or presentation of results.	p7-8
Synthesis methods	13a	Describe the processes used to decide which studies were eligible for each synthesis (e.g. tabulating the study intervention characteristics and comparing against the planned groups for each synthesis (item #5)).	p7-8, Suppl. p9-12, Tables 1 and 2
	13b	Describe any methods required to prepare the data for presentation or synthesis, such as handling of missing summary statistics, or data conversions.	p7-8, Suppl. p10-12
	13c	Describe any methods used to tabulate or visually display results of individual studies and syntheses.	p7-8, Suppl. p10-12
	13d	Describe any methods used to synthesize results and provide a rationale for the choice(s). If meta-analysis was performed, describe the model(s), method(s) to identify the presence and extent of statistical heterogeneity, and software package(s) used.	p7-8, Suppl. p10-12
	13e	Describe any methods used to explore possible causes of heterogeneity among study results (e.g. subgroup analysis, meta-regression).	p7-8, Suppl. p10-12
	13f	Describe any sensitivity analyses conducted to assess robustness of the synthesized results.	p7-8, Suppl. p10-12
Reporting bias assessment	14	Describe any methods used to assess risk of bias due to missing results in a synthesis (arising from reporting biases).	p7-8, Suppl. p10-12

Section and Topic	Item #	Checklist item	Location where item is reported
Certainty assessment	15	Describe any methods used to assess certainty (or confidence) in the body of evidence for an outcome.	p8
**RESULTS**			
Study selection	16a	Describe the results of the search and selection process, from the number of records identified in the search to the number of studies included in the review, ideally using a flow diagram.	Suppl. Figure 1, p8-9
	16b	Cite studies that might appear to meet the inclusion criteria, but which were excluded, and explain why they were excluded.	p8-9, Suppl. Table 3
Study characteristics	17	Cite each included study and present its characteristics.	p8-9, Tables 1-2, Suppl. Table 3
Risk of bias in studies	18	Present assessments of risk of bias for each included study.	p13, Suppl. Tables 4-5
Results of individual studies	19	For all outcomes, present, for each study: (a) summary statistics for each group (where appropriate) and (b) an effect estimate and its precision (e.g. confidence/credible interval), ideally using structured tables or plots.	p9-13, Figures 1-6, Suppl. Table 3, Suppl. Figures 2-31
Results of syntheses	20a	For each synthesis, briefly summarise the characteristics and risk of bias among contributing studies.	p9-13, Suppl. Tables 4-6, Figures 1-6, Suppl. Figures 2-31

Section and Topic	Item #	Checklist item	Location where item is reported
	20b	Present results of all statistical syntheses conducted. If meta-analysis was done, present for each the summary estimate and its precision (e.g. confidence/credible interval) and measures of statistical heterogeneity. If comparing groups, describe the direction of the effect.	p9-13, Figures 1-6, Suppl. Figures 2-31
	20c	Present results of all investigations of possible causes of heterogeneity among study results.	
	20d	Present results of all sensitivity analyses conducted to assess the robustness of the synthesized results.	Suppl. p55
Reporting biases	21	Present assessments of risk of bias due to missing results (arising from reporting biases) for each synthesis assessed.	p13, Suppl. Table 4-5
Certainty of evidence	22	Present assessments of certainty (or confidence) in the body of evidence for each outcome assessed.	p13, Suppl. Table 6
**DISCUSSION**			
Discussion	23a	Provide a general interpretation of the results in the context of other evidence.	p13-16
	23b	Discuss any limitations of the evidence included in the review.	p17
	23c	Discuss any limitations of the review processes used.	p17
	23d	Discuss implications of the results for practice, policy, and future research.	p17-18
**OTHER INFORMATION**			
Registration and protocol	24a	Provide registration information for the review, including register name and registration number, or state that the review was not registered.	p5
	24b	Indicate where the review protocol can be accessed, or state that a protocol was not prepared.	p5

Section and Topic	Item #	Checklist item	Location where item is reported
	24c	Describe and explain any amendments to information provided at registration or in the protocol.	p5
Support	25	Describe sources of financial or non-financial support for the review, and the role of the funders or sponsors in the review.	p26
Competing interests	26	Declare any competing interests of review authors.	p27
Availability of data, code and other materials	27	Report which of the following are publicly available and where they can be found: template data collection forms; data extracted from included studies; data used for all analyses; analytic code; any other materials used in the review.	p19

Supplementary Table 1. PRISMA 2020 Checklist

From: Page MJ, McKenzie JE, Bossuyt PM, Boutron I, Hoffmann TC, Mulrow CD, et al. The PRISMA 2020 statement: an updated guideline for reporting systematic reviews. BMJ 2021;372:n71. doi: 10.1136/bmj.n71

2. Predefined outcomes

We defined our primary outcomes in advance as in-hospital mortality; presence of intraventricular hemorrhage (IVH) (all grades and severe (≥ grade 3)), periventricular leukomalacia and cerebral palsy.

The predefined secondary outcomes were: oxygen saturation level by pulse oximetry (SpO_2), heart rate (HR) and Apgar score at 1, 5 and 10 minutes after birth; presence of bronchopulmonary dysplasia (BPD), patent ductus arteriosus (PDA), necrotizing enterocolitis (NEC), retinopathy of prematurity (ROP); number of blood transfusions; need for blood transfusion, phototherapy, surfactant therapy; time from birth to first cry and to initiate respiratory support; maximum fraction of inspired oxygen (FiO_2); hematocrit level within 24 hours of life; umbilical cord pH; polycythaemia; early and late onset neonatal sepsis (EOS, LOS); temperature at admission to the neonatal intensive care unit (NICU); hypothermia (<36.0°C); maternal outcomes such as blood loss, postpartum (pp.) infection and hemorrhage

Primary predefined outcomes **we could not perform analysis of**: periventricular leukomalacia, cerebral palsy

Secondary predefined outcomes **we could not perform analysis of**: oxygen saturation level at 1 minute after birth, Apgar score at 10 minutes after birth, heart rate at 1, 5 and 10 minutes after birth, number of blood transfusions, time from birth to first cry, time from birth to initiate respiratory support, maximum fraction of inspired oxygen, hematocrit level within 24 hours of birth, umbilical cord pH, polycythaemia, early onset neonatal sepsis, hypothermia (<36 Celsius), postpartum infection, postpartum hemorrhage, maternal blood loss

3. Details of our outcomes

Analysed outcomes	Inclusion criteria for analysis
In-hospital mortality	mortality rate before discharge from hospital
All grades of IVH	grade 1-4 assessed by Papile classification
Severe IVH	≥ grade 3 assessed by Papile classification
BPD	chronic lung disease, requirement for respiratory support or supplementary oxygen at 36 weeks postmentrual age
PDA requiring treatment	PDA requiring treatment
NEC ≥ stage 2	≥ stage 2 assessed by Bell staging
ROP requiring treatment	ROP requiring treatment
Need for blood transfusion	requiring blood transfusion
Need for phototherapy	requiring phototherapy
Need for surfactant therapy	requiring surfactant therapy
LOS	occured after 72 hours of life
SpO_2 at 5 and 10 minutes after birth	readings from pulse oximeter
Apgar score at 1, 5 and 10 minutes after birth	assessed by a health care worker
Temperature at admission to the NICU	temperature assessed by a health care worker at the NICU admission

Supplementary Table 2. Inclusion criteria of our outcomes for analysis based on the included studies

IVH: intraventricular hemorrhage, BPD: bronchopulmonary dysplasia, PDA: patent ductus arteriosus, NEC: necrotizing enterocolitis, ROP: retinopathy of the prematurity, LOS: late-onset neonatal sepsis, SpO_2: arterial oxygen saturation level, NICU: neonatal intensive unit care

4. Predefined search key

("intact cord" OR "sustained cord" OR "cord clamping" OR "intact placental circulation" OR "sustained cord circulation" OR "umbilical cord" OR "uncut umbilical cord" OR "clamped cord")

AND

(resuscitation OR "active care" OR "delivery room care" OR "delivery room management" OR "airway opening manoeuvres" OR "airway opening maneuvres" OR "positive pressure ventilation" OR "positive pressure ventillation" OR intubation OR "chest compression" OR "respiratory support" OR stabilisation OR stabilization OR ventilation OR ventillation OR reamination OR "life support" OR management OR "not vigorous" OR "non vigorous" OR nonvigorous OR non-vigorous OR "not crying" OR "non crying" OR noncrying OR non-crying OR "not breathing" OR "non breathing" OR nonbreathing OR non-breathing OR CPAP OR "continuous positive airway pressure")

5. Detailed description of the statistical analysis

We provide the following additional details on data synthesis.

For mean difference (MD) effect size calculations, in cases if instead of the mean, standard deviation (SD), the quartiles were given, for estimating the mean and standard deviation from the quartiles Lou[1] and Shi[2] methods were used (as implemented in the used *meta* R package). As a limitation, we should highlight that this is an estimation. Although, based on the other publications in the literature about these outcomes (temperature, arterial oxygen saturation level (SpO_2)) and the data in the used publication, we could assume that the distribution of these variables is not relevantly differ from a normal or log-normal distribution, therefore this conservative estimation might give only small bias in the point estimates, but with larger confidence interval (CI). In case of Apgar score, nearly all study give the effect with quartiles

that assumes not normal distribution. Based on our data (not reported), we see a left skewed distribution. Unfortunately nor the logarithmic, square-root or cubic-root transformation helped to create normal distribution (based on plots). As we do not know the distribution, we could not estimate well the median and quartiles for the study where the result is given in mean and SD, therefore this study was excluded from this analysis for a better estimation. For calculating the CI of the (estimated) difference between group medians, the quantile estimation method was used. We used the quantile estimation in each study based on the best fitting normal, log-normal, gamma, Weibull distribution as referred S2 scenario in McGrath's article[3]. Apgar score results are more questionable as the values shows very small variability in our studies (in some cases, the different quartiles were equal). Additionally, as sensitivity analysis, we performed an analysis mentioned for temperature and SpO_2.

For pooling the effect size, pooled risk ratio (RR) was calculated by the Mantel-Haenszel method[4,5]. Exact Mantel-Haenszel method (without continuity correction) was used to handle zero cell counts (as recommended by Cooper, Hedges, and Valentine[6]; J. Sweeting, J. Sutton, and C. Lambert[7]). Inverse variance weighting method was used to calculate the pooled MD. We used a Hartung-Knapp adjustment[8,9] for CIs. This adjustment was applied only if it is more conservative then the classical one (as recommended by Jackson et al.[10] as hybrid method 2).

To estimate the heterogeneity variance measure (τ^2), for RR calculation the Paule-Mandel method[11] (recommended by Veroniki et al.[12]), for MD and median difference (MedD), the restricted maximum-likelihood estimator was used with the Q profile method for confidence interval Veroniki et al.[12].

On the forest plots in case of 0 cell counts, individual study RR with 95% CI was calculated by adding 0.5 as continuity correction (it was used only for visualization on forest plot). The t-distribution based method used for CI of MD and MedD calculation of individual studies.

In case of subgroup analysis we used a fixed-effects "plural" model (aka. mixed-effects model). We assumed that all subgroup share a common τ^2 value as we did not anticipate difference in the between-study heterogeneity in the subgroups and the study number was relatively small in some subgroup (recommended in Borenstein et al.[13]).

The subgroup analysis was planned before the data extraction in case of all variables, except subgroup analysis based on the usage of special resuscitation trolley.

6. PRISMA 2020 flowchart

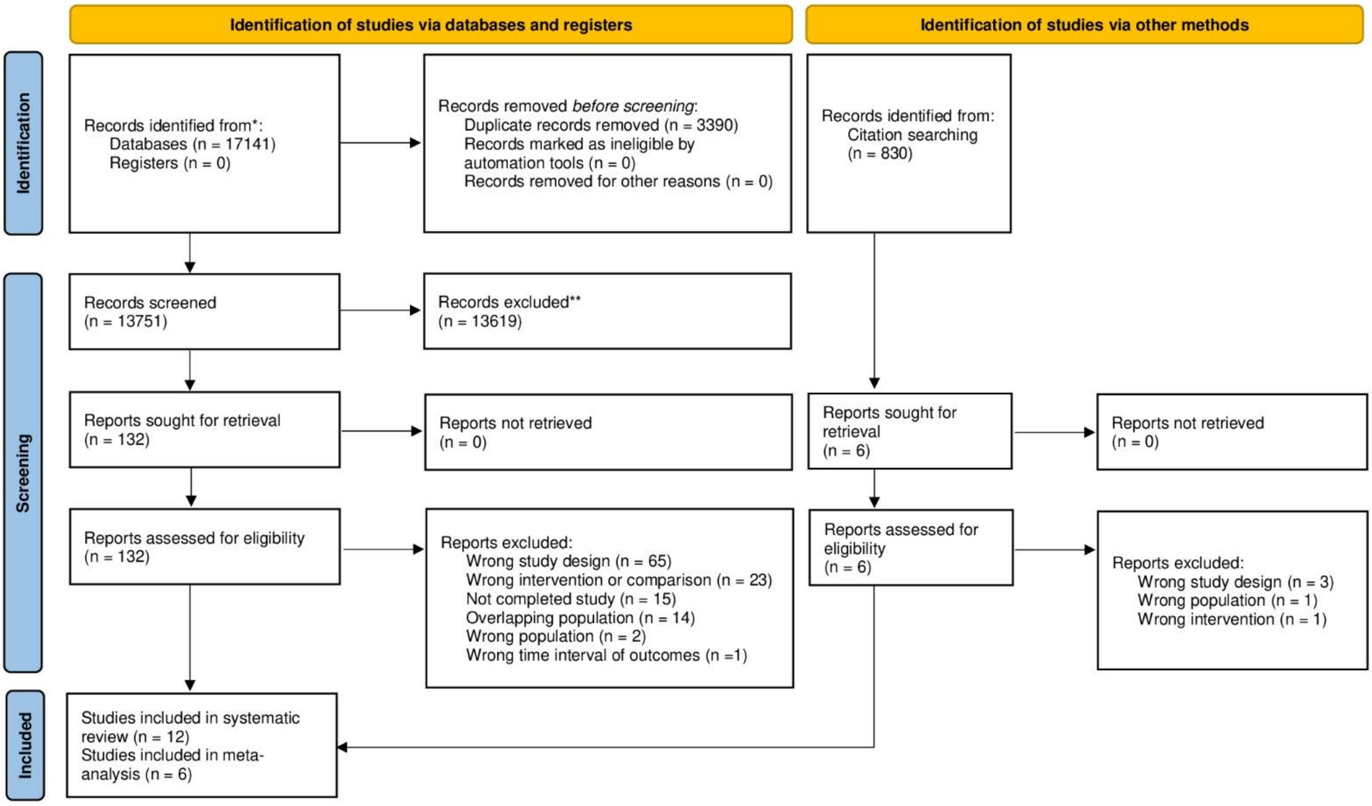

Supplementary Figure 1. PRISMA 2020 flowchart representing the study selection process

Page MJ, McKenzie JE, Bossuyt PM, Boutron I, Hoffmann TC, Mulrow CD, et al. The PRISMA 2020 statement: an updated guideline for reporting systematic reviews. BMJ 2021;372:n71. doi: 10.1136/bmj.n71.

7. Details and results of studies we included in the systematic review

First author and year of publication	Country	Study type	Population	Intervention group (I)	Control group (C)	Results included in the systematic review (dichotomous outcomes: proportion %, continuous outcomes: mean (SD), median [IQR: Q1, Q3], median {range}, MD (95% CI), MedD (95% CI))	Reason of exclusion from analysis
**Badurdeen 2022**[14]	Australia	RCT	infants ≥32 weeks of GA	n=63<br><br>PBCC: establishment of effective pulmonary gas exchange, either via PPV or effective spontaneous breathing, prior to umbilical cord clamping	n=60<br><br>ECC: immediate cord clamping followed by resuscitation	**HR between 60 to 120 sec after birth (bpm):** MD –6 (–17, 5)<br>**Apgar score at 1 min:** I: **7** [5, 8], C: 7 [6, 9]<br>**Apgar score at 5 min:** I: 9 [9, 9], C: 9 [9, 9]<br>**Apgar score at 10 min:** I: 10 [9, 10] n=41, C: 10 [10, 10] n=37<br>**Phototherapy**: I: 16%, C: 12%<br>**Maternal blood loss (ml):** I: 469 (326), C: 518 (573)<br>**Postpartum hemorrhage 500-999 ml:** I: 26%, C: 23%<br>**Postpartum hemorrhage ≥1000 ml:** I: 8%, C: 8%<br>**Maternal infection (postpartum):** I: 3%, C: 0%	study population including infants for whom it was either uncertain or unnecessary to administer any form of resuscitation following birth

**Deng 2022**[15]	China	RCT	preterm infants <32 weeks of GA	n=80<br><br>DCC+nCPAP: at least 60 sec of DCC with nCPAP	n=80<br><br>DCC-only: at least 60 sec of DCC without nCPAP	**Death during hospitalization:** I: 2.50%, C: 3.75%<br>**SpO_2 at 5 min (%):** MedD: 0.00 (–2.00, 1.00)<br>**SpO_2 at 10 min (%):** MedD: 0.00 (–1.00, 1.00)<br>**Apgar score at 1 min:** I: 9.00 [8.00, 10.00], C: 9.00 [8.00, 10.00], MedD: 0.00 (–1.00, 0.00)<br>**Apgar score at 5 min:** I: 9.00 [9.00, 10.00], C: 10.00 [9.00, 10.00], MedD: 0.00 (0.00, 0.00)<br>**Apgar score at 10 min:** I: 10.00 [9.00, 10.00], C: 10.00 [9.00, 10.00], MedD: 0.00 (0.00, 0.00)<br>**Admission temperature:** I: 36.96 (0.35), C: 36.95 (0.33), MD: 0.13 (–0.09, 0.12)<br>**IVH ≥ grade 3:** I: 1.25%, C: 1.25%<br>**NEC ≥ phase 2:** I: 6.25%, C: 2.50%<br>**ROP ≥ phase 2:** I: 11.25%, C: 8.75%<br>**BPD all grades:** I: 2.50%, C: 10.00%<br>**LOS:** I: 1.25%, C: 3.75%<br>**Admission temperature <36.0 Celsius (rectal):** I: 0, C: 0<br>**Postpartum hemorrhage ≥1000 ml:** I: 2.5%, C: 2.5%<br>**Postpartum infection:** I: 2.5%, C: 2.5%	study population including infants for whom it was either uncertain or unnecessary to administer any form of resuscitation following birth

**Duley 2018**[16]	UK	RCT	preterm infants <32 weeks of GA	n=135<br><br>umbilical cord clamping after at least 2 min and, if needed, immediate neonatal stabilisation and resuscitation with cord intact	n=134<br><br>clamping within 20 sec and, if needed, immediate neonatal stabilisation and resuscitation after clamping	**Death before discharge:** I: 5%, C: 11%<br>**Temperature at admission to the NICU:** I: 36.7 (0.6), C: 36.9 (0.8)<br>**Any IVH (grade 1-4):** I: 32%, C: 36%<br>**Severe IVH (≥ grade 3):** I: 4%, C: 5%<br>**NEC ≥ grade 2:** I: 6%, C: 4%<br>**ROP requiring treatment:** I: 4%, C: 4%<br>**Chronic lung disease:** I: 31%, C: 33%<br>**PDA requiring treatment:** I: 15%, C: 15%<br>**Blood transfusion (any):** I: 47%, C: 52%<br>**Phototherapy:** I: 92%, C: 91%<br>**Temperature at admission to the NICU ≤ 36 Celsius:** I: 13%, C: 11%<br>**Blood loss at birth ≥500 ml:** I: 45%, C: 48%<br>**Blood loss at birth ≥1000 ml:** I: 8%, C: 10%<br>**Postpartum infection+parenteral antibiotics:** I: 26%, C: 23%	study population including infants for whom it was either uncertain or unnecessary to administer any form of resuscitation following birth
**Katheria 2017**[17]	USA	RCT		n=30	n=30	**Apgar score at 1 min:** I: 8 [9, 9], C: 8 [8, 8]	

			neonates ≥37 weeks of GA at risk for resuscitation	1-min group: umbilical cord clamped and cut by 1 min and were placed either on the mother's abdomen or, if depressed the cord was cut immediately (within seconds), they were transitioned to the radiant warmer, resuscitation was defined as receiving stimulation to breathe, blow by oxygen, or positive pressure ventilation by endotracheal tube or mask	5-min group: placed on the mother's abdomen if vigorous, otherwise, they were placed on the LifeStart bed, which was equipped with resuscitation tools, resuscitation interventions (warm dry and stimulate initially if apneic, oxygen and/or ventilation if needed based on spontaneous breathing, SpO2, or HR) were equivalent to the 1-min group, if the infant did not have adequate breathing (continued need for ventilation or oxygen), clamping was delayed	**Apgar score at 5 min:** I: 9 [9, 9], C: 8 [8, 8]<br><br>There were no differences between groups in HR or SpO_2 for the first 5 min in the overall group, but infants undergoing 5-min DCC who did not receive supplemental oxygen or PPV had greater SpO_2 levels at 3 and 4 min of life ($p = 0.03$).	study population including infants for whom it was either uncertain or unnecessary to administer any form of resuscitation following birth

					past the 5-min cutoff until the infant transitioned (stable HR and SpO2), the cord stopped pulsating, or the placenta was delivered		
**Hocq 2022**[18]	Belgium	descriptive observational study - cohort	infants <32 weeks of GA	n=18<br><br>ICR cohort:<br>ICR with DCC using the LifeStart trolley, clamp the cord when the best achievable lung aeration and breathing were reached, cord clamping prior to 2 min was considered DCC failure	n=30<br><br>pre-ICR/historical cohort: 9-month period preceding ICR, managed with ICC	**Death before discharge from NICU:** I: 11.11%, C: 16.67%<br>**Apgar score at 1 min:** I: 4.5 [3.75, 6.25], C: 6 [4.75, 7.25]<br>**Apgar score at 5 min:** I: 8 [6.75, 9], C: 8 [8, 9]<br>**Apgar score at 10 min:** I: 9 [8, 9], C: 9 [8, 9]<br>**Temperature at admission to the NICU:** I: 36.6 [35.8, 36.9], C: 36.7 [36.4, 37.02]<br>**Any IVH (grade 1-4):** I: 19%, C: 40%<br>**NEC ≥ grade 2:** I: 0%, C: 12%<br>**ROP treated by laser therapy:** I: 6%, C: 4%<br>**Need of non-invasive respiratory support or oxygen-therapy at 36 weeks:** I: 19%, C: 24%<br>**PDA requiring treatment:** I: 19%, C: 16%<br>**LOS:** I: 6%, C: 20%	study type (observational study)

						**Phototherapy rate:** I: 81%, C: 88%<br>**Surfactant administration:** I: 39%, C: 40%<br>**Moderate hypothermia on admission (32-35.9 Celsius):** I: 22%, C: 7%<br>**Mild hypothermia on admission (36-36.5 Celsius):** I: 16%, C: 20%	
**Hoeller 2024**[19]	Austria	observational study (retrospective)	VLBW infants born at <32+0 weeks of gestation	n=27<br><br>PBCC group:<br>neonates were placed in a sterile plastic bag on the resuscitation platform of the Concord Birth Trolley immediately after birth, and an overhead heater was positioned right above the platform to prevent heat loss, tactile	n=27<br><br>control group:<br>the cord was routinely clamped 30–60 sec after birth, postnatal stabilisation and resuscitation were performed in the Giraffe incubator or on the CosyCot resuscitation desk, the heated Neopuff Infant T-Piece	**In-hospital mortality:** I: 7%, C: 7%<br>**Apgar score at 1 min:** I: 8 {2-8}, C: 7 {4-9}<br>**Apgar score at 5 min:** I: 8 {6-9}, C: 8 {6-9}<br>**Apgar score at 10 min:** I: 9 {6-10}, C: 9 {7-10}<br>**Significant differences in HR in the first 72 h after birth (bpm):**<br>• **14 h: I: 141 (11), C: 146 (9)**<br>• **15 h: I: 140 (11), C: 147 (10)**<br>• **16 h: I: 140 (11), C: 147 (11)**<br>• **18 h: I: 142 (12), C: 149 (11)**<br>• **27 h: I: 143 (10), C: 150 (12)**<br>• **34 h: I: 145 (7), C: 151 (13)**<br>• **50 h: I: 149 (7), C: 155 (12)**	study type (observational study)

				stimulation was performed before respiratory support was started on non-vigorous preterm neonates, depending on the infant's respiratory effort, either CPAP ventilation or IPPV was administered via a face mask, cord clamping was performed if the neonate demonstrated sufficient spontaneous breathing and a constant increase in SpO2 (sufficient spontaneous breathing was defined as regular thoracic excursions, assisted by respiratory support with	Resuscitator was used to provide respiratory support, the neonates were also placed in a sterile plastic bag during postnatal stabilisation, and an overhead warmer was placed above the neonate to prevent loss of temperature	• **57 h: I: 149 (9), C: 157 (12)**<br>• **58 h: I: 150 (9), C: 157 (11)**<br>• **59 h: I: 150 (9), C: 157 (10)**<br>**IVH all grades:** I: 22%, C: 22%<br>• **IVH I°:** I: 11%, C: 15%<br>• **IVH II°:** I: 0%, C: 0%<br>• **IVH III°:** I: 4%, C: 4%<br>**NEC: I: 7%, C: 15%**<br>**ROP all grades:** I: 30%, C: 33%<br>• **ROP I°:** I: 7%, C: 22%<br>• **ROP II°:** I: 15%, C: 7%<br>• **ROP III°:** I: 7%, C: 4%<br>**BPD all grades: I: 19%, C: 15%**<br>**LOS: I: 15%, C: 11%**	

				CPAP ventilation and/or short periods of IPPV)			
**Andersson 2019**[20]	Nepal	RCT	infants ≥33 weeks of GA in need of resuscitation	n=74<br><br>resuscitation with an intact umbilical cord close to the mother in her bed, delay in cord clamping was instructed to be at least 180 sec in the intact cord group	n=48<br><br>standard care (resuscitation according to the HBB algorithm including ECC) at a designated area, on a resuscitation table in a room next to the delivery room	**HR at 1 min (bpm):** MD –10 (–11, –8)<br>**HR at 5 min (bpm):** MD –10 (–11, –8)<br>**HR at 10 min (bpm):** MD 1 (0, 2)	-
**Knol 2020**[21]	The Netherlands	RCT	infants <32 weeks of GA	n=20<br><br>preterms were stabilised according to the PBCC	n=17<br><br>standard DCC group were transferred to the standard	**Temperature at admission to the NICU <36 Celsius:** I: 20%, C: 5.9%<br>**Maternal blood loss (ml):** I: 300 [200, 700], C: 450 [263, 538]	-

				approach: providing standard postnatal respiratory management and heat loss prevention while the infant was on the Concord close to its mother, with the cord still intact, which was clamped only after the infant was judged to be stable, as defined by the presence of regular spontaneous breathing, a HR >100 bpm and SpO2 above 90% while using FiO_2 <0.40	resuscitation table following cord clamping (30-60 sec) to administer any treatments or interventions required to stabilise the infant, according to local resuscitation guidelines	**Postpartum hemorrhage (>1000 ml):** I: 11.1%, C: 12.5%	
**Nevill 2022**[22]	New Zealand	RCT	preterm infants less than 31 weeks of gestation	n=57	n=56<br><br>comparison	**Maternal blood loss (ml):** I: 400 [300, 1000], C: 400 [300, 587]<br>**Postpartum hemorrhage (≥500 ml):** I: 50%, C: 41%	-

			undergoing DCC, provided they were either not breathing or making irregular nonsustained breathing efforts during DCC	intervention group was given PPV using a T piece resuscitator with colorimetric carbon dioxide detection device, a PIP of 20 cm water and PEEP of 5 cm with 10 L of gas flow and blended oxygen of 30% was delivered for 30 sec and the cord clamped at 50 sec	group continued to receive gentle stimulation and the cord was also clamped at 50 sec	**Infection received >48 hours antibiotics first week postpartum:** I: 7%, C: 12.5%	
**Raina 2022**[23]	India	RCT	infants born at ≥34 weeks of gestation to women with pregnancy or labor	n=71<br><br>the neonates were received in a sterile prewarmed sheet on the special resuscitation	n=91<br><br>the cord was clamped within 30 sec of birth, and resuscitation was initiated	**HR at 1 min (bpm):** MD 4.3 (–4.1, 12.7)<br>**HR at 5 min (bpm):** MD –11.5 (–16.0, –7.1)<br>**HR at 10 min (bpm):** MD –20.1 (–24.5, –15.8)	-

			complications and requiring resuscitation at birth	trolley, PPV was provided with a T-piece resuscitator while keeping the umbilical cord intact, the cord was clamped after at least 180 sec or when the neonate exhibited spontaneous breathing, whichever was later, with a maximum time limit of 5 min	at the standard resuscitation corner		

Supplementary Table 3. Details and results of studies we included in the systematic review

I: intervention group, C: control group, SD: standard deviation, IQR: interquartile range, Q1: first or lower quartile, Q3: third or upper quartile, MD: mean difference, CI: confidence interval, MedD: median difference, RCT: randomized controlled trial, GA: gestational age, n: number, PBCC: physiological-based cord clamping, PPV: positive pressure ventilation, ECC: early cord clamping, HR: heart rate, sec: second(s), bpm: beat per minute, min: minute(s), ml: mililiter, DCC: delayed cord clamping, nCPAP: nasal continuous positive airway pressure, SpO_2: peripheral arterial oxygen saturation, IVH: intraventricular

hemorrhage, NEC: necrotizing enterocolitis, ROP: retinopathy of the prematurity, BPD: bronchopulmonary dysplasia, LOS: late-onset neonatal sepsis, UK: United Kingdom, NICU: neonatal intensive care unit, PDA: patent ductus arteriosus, USA: United States of America, ICR: intact cord resuscitation, ICC: immediate cord clamping, VLBW: very low birth weight (<1500 g), CPAP: continuous positive airway pressure, IPPV: intermittent positive pressure ventilation, h: hours, I°: grade I, II°: grade II, III°: grade III, HBB: Helping Babies Breathe, FiO2: fraction of inspired oxygen, PIP: peak inspiratory pressure, cm: centimeter(s), PEEP: positive end-expiratory pressure, L: liter(s)

8. Additional results

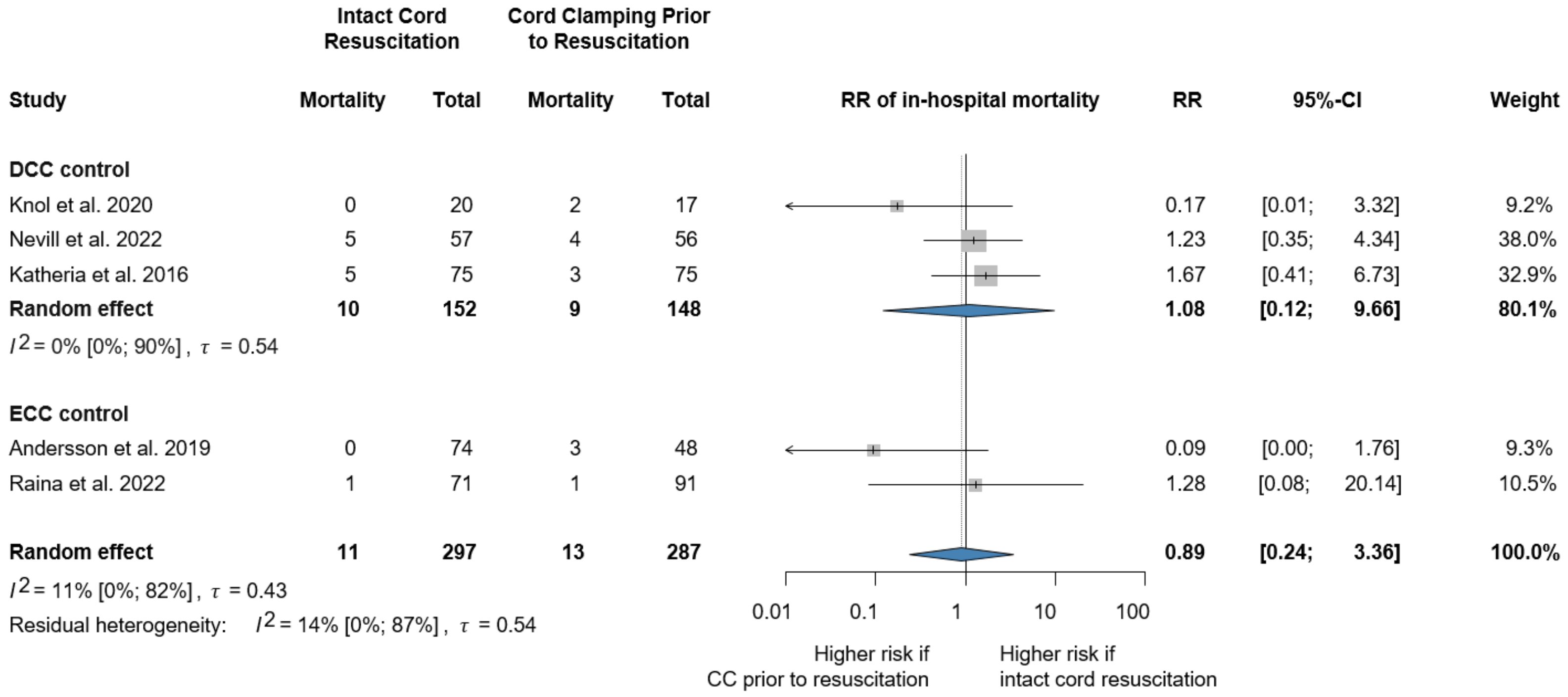

Supplementary Figure 2. Forest plot representing the risk ratio of in-hospital mortality in DCC vs. ECC control subgroups among infants who received intact cord resuscitation or cord clamping prior to resuscitation after birth

DCC: delayed cord clamping, ECC: early cord clamping, RR: risk ratio, 95%-CI: 95% confidence interval, CC: cord clamping

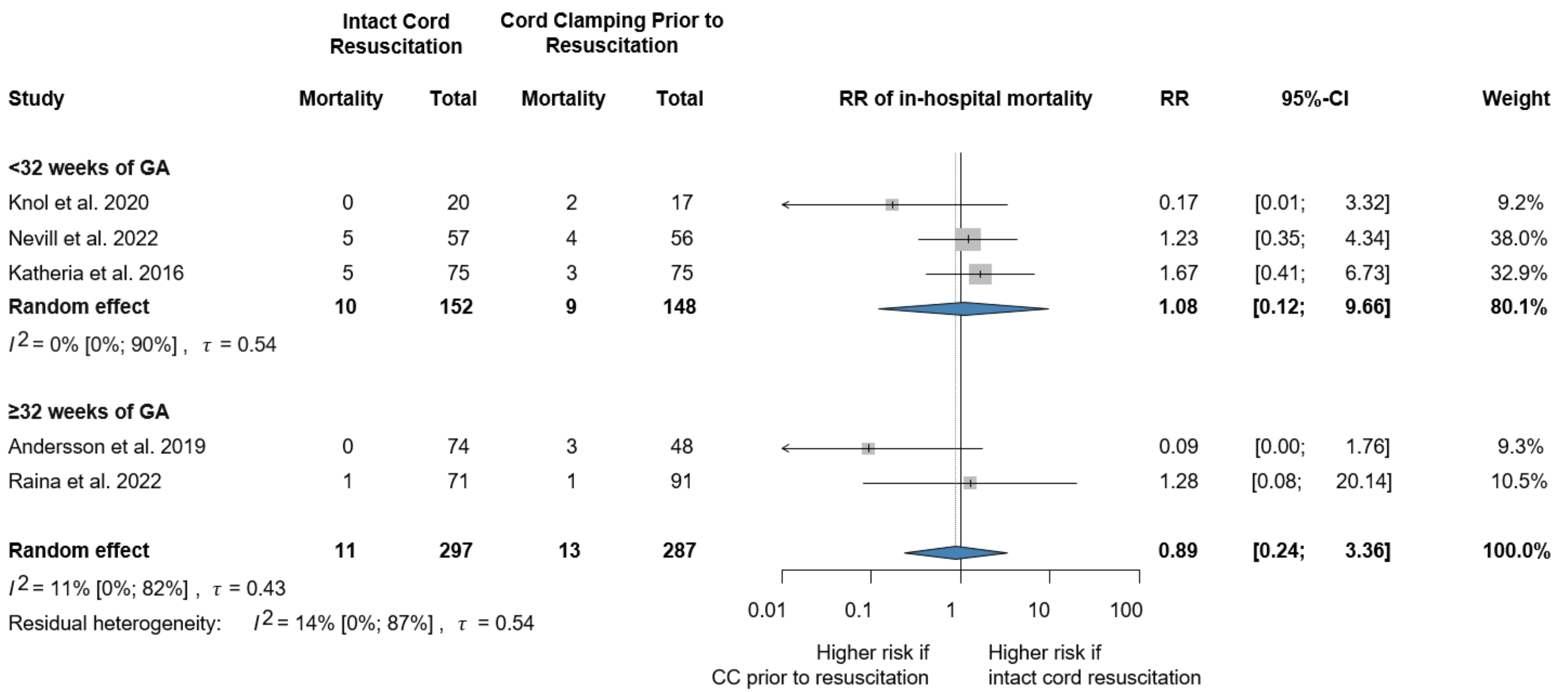

Supplementary Figure 3. Forest plot representing the risk ratio of in-hospital mortality in infants <32 and ≥32 weeks of GA who received intact cord resuscitation or cord clamping prior to resuscitation after birth

GA: gestational age, RR: risk ratio, 95%-CI: 95% confidence interval, CC: cord clamping

Study	Intact Cord Resuscitation: Mortality	Intact Cord Resuscitation: Total	Cord Clamping Prior to Resuscitation: Mortality	Cord Clamping Prior to Resuscitation: Total	RR	95%-CI	Weight
**with special resuscitation trolley**							
Knol et al. 2020	0	20	2	17	0.17	[0.01; 3.32]	9.2%
Raina et al. 2022	1	71	1	91	1.28	[0.08; 20.14]	10.5%
Katheria et al. 2016	5	75	3	75	1.67	[0.41; 6.73]	32.9%
**Random effect**	**6**	**166**	**6**	**183**	**0.96**	**[0.06; 15.21]**	**52.7%**
I^2 = 0% [0%; 90%] , τ = 0.80							
**without special resuscitation trolley**							
Andersson et al. 2019	0	74	3	48	0.09	[0.00; 1.76]	9.3%
Nevill et al. 2022	5	57	4	56	1.23	[0.35; 4.34]	38.0%
**Random effect**	**11**	**297**	**13**	**287**	**0.89**	**[0.24; 3.36]**	**100.0%**
I^2 = 11% [0%; 82%] , τ = 0.43							
Residual heterogeneity: I^2 = 31% [0%; 75%] , τ = 0.80							

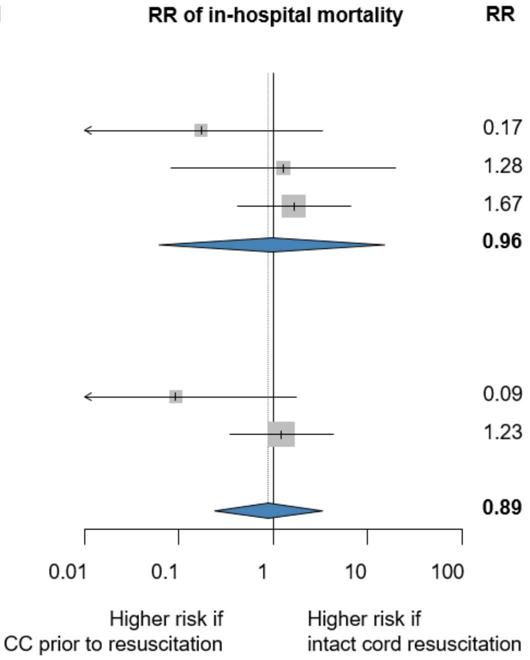

Supplementary Figure 4. Forest plot representing the risk ratio of in-hospital mortality in infants who received intact cord resuscitation (with or without special resuscitation trolley) or cord clamping prior to resuscitation after birth

RR: risk ratio, 95%-CI: 95% confidence interval, CC: cord clamping

Study	Intact Cord Resuscitation				Cord Clamping Prior to Resuscitation				MedD	95%-CI	Weight
	Total	Median	Q1	Q3	Total	Median	Q1	Q3			
Knol et al. 2020	20	6	5	8	17	7	6.00	8	-1.00	[-2.51; 0.51]	9.0%
Finn et al. 2019	14	6	5	7	12	6	4.30	8	0.00	[-2.17; 2.17]	4.4%
Katheria et al. 2016	63	7	4	7	62	7	5.00	7	0.00	[-0.83; 0.83]	30.4%
Nevill et al. 2022	57	5	3	6	56	5	4.00	7	0.00	[-1.03; 1.03]	19.6%
Raina et al. 2022	71	4	2	5	91	4	3.00	5	0.00	[-0.75; 0.75]	36.6%
**Random effect**									**-0.09**	**[-0.55; 0.36]**	**100.0%**

$I^2 = 0\%$ [0%; 79%] , $\tau = 0$

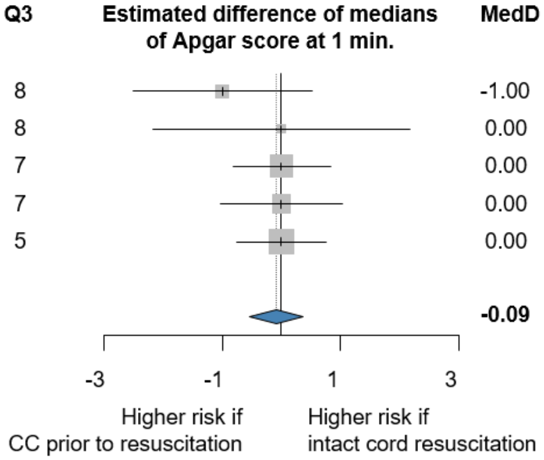

Supplementary Figure 5. Forest plot representing the estimated MedD of Apgar score at 1 minute after birth in infants who received intact cord resuscitation or cord clamping prior to resuscitation after birth

MedD: median difference, Q1: first or lower quartile, Q3: third or upper quartile, min: minute, 95%-CI: 95% confidence interval, CC: cord clamping

Study	Intact Cord Resuscitation Total	Median	Q1	Q3	Cord Clamping Prior to Resuscitation Total	Median	Q1	Q3	MedD	95%-CI	Weight
Knol et al. 2020	20	8	7	9	17	9	8	9	-1.00	[-1.93; -0.07]	3.2%
Katheria et al. 2016	63	8	7	8	62	8	7	8	0.00	[-0.33; 0.33]	25.8%
Nevill et al. 2022	57	8	6	9	56	8	6	9	0.00	[-1.02; 1.02]	2.7%
Raina et al. 2022	71	9	9	9	91	9	8	9	0.00	[-0.20; 0.20]	68.3%
**Random effect**									**-0.03**	**[-0.36; 0.29]**	**100.0%**

I^2 = 30% [0%; 75%], τ < 0.01

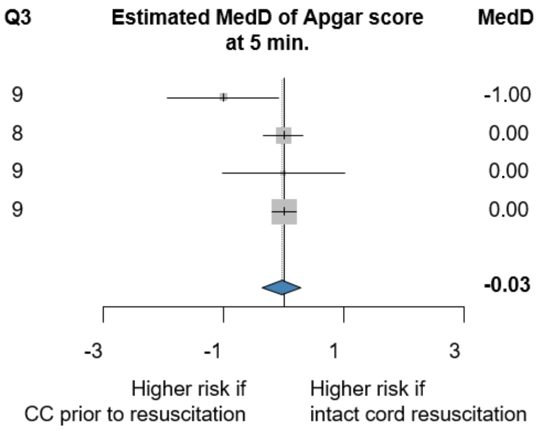

Supplementary Figure 6. Forest plot representing the estimated MedD of Apgar score at 5 minutes after birth in infants who received intact cord resuscitation or cord clamping prior to resuscitation after birth

MedD: median difference, Q1: first or lower quartile, Q3: third or upper quartile, min: minutes, 95%-CI: 95% confidence interval, CC: cord clamping

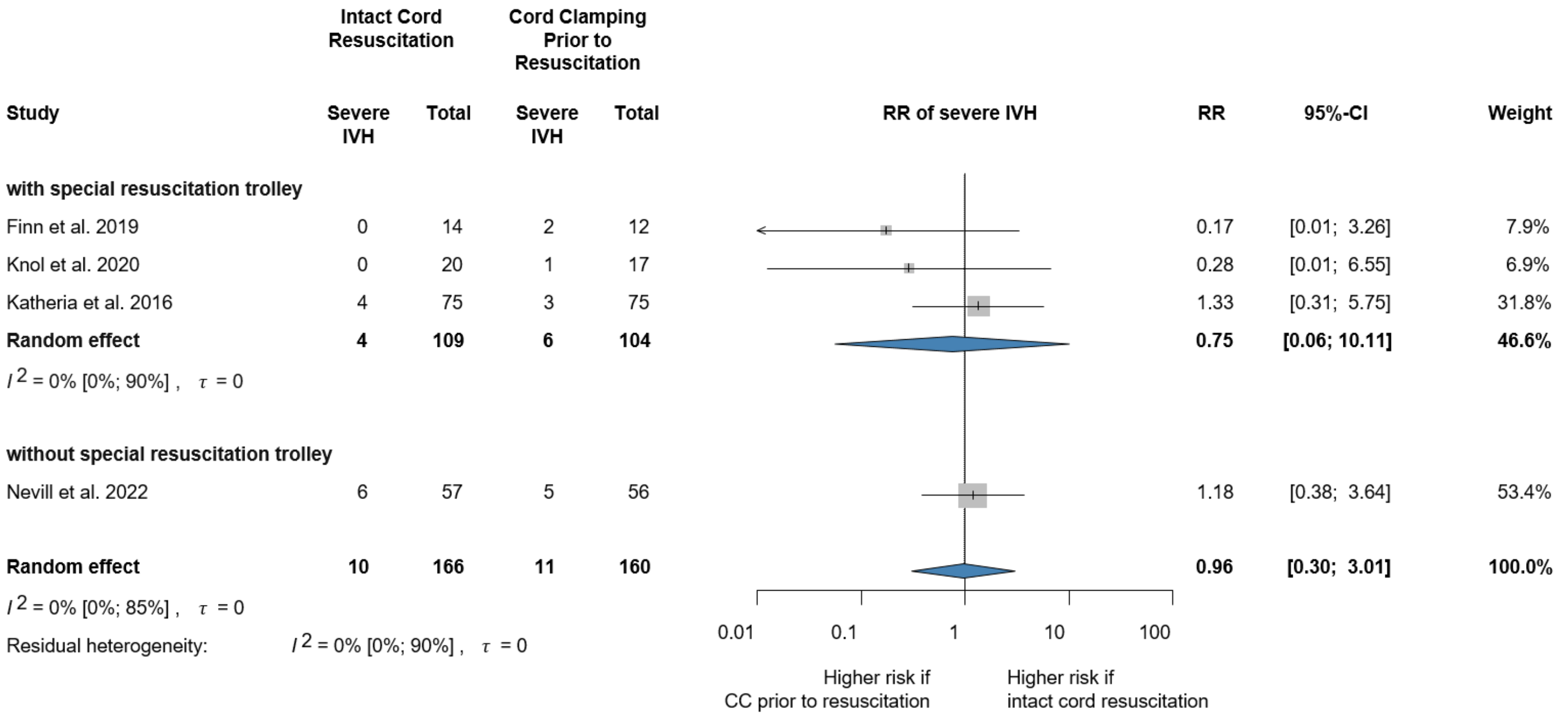

Supplementary Figure 7. Forest plot representing the risk ratio of severe IVH (≥ grade 3) in infants who received intact cord resuscitation (with or without special resuscitation trolley) or cord clamping prior to resuscitation after birth

IVH: intraventricular hemorrhage, RR: risk ratio, 95%-CI: 95% confidence interval, CC: cord clamping

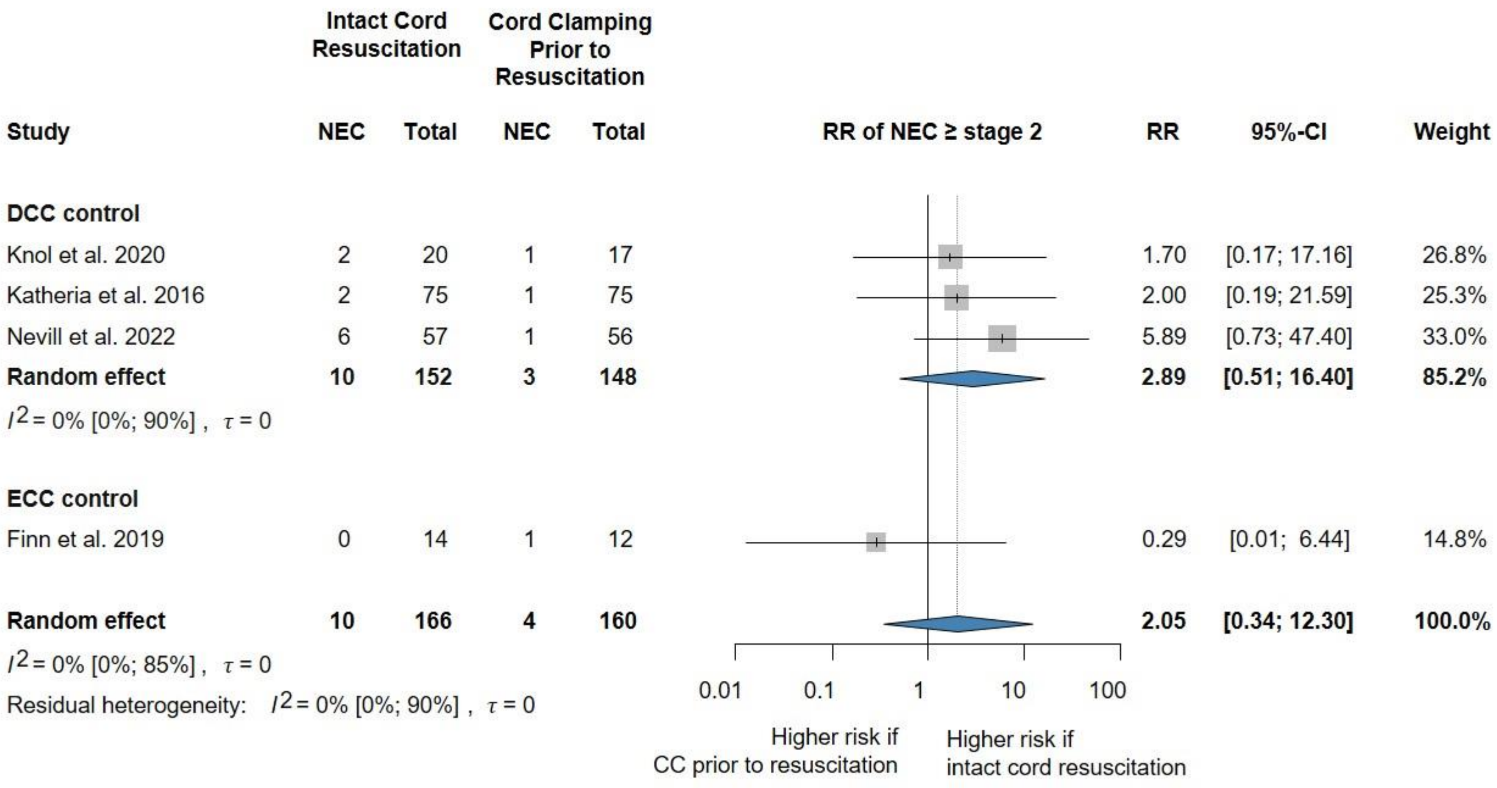

Supplementary Figure 8. Forest plot representing the risk ratio of NEC ≥ stage 2 in DCC vs. ECC control subgroups among infants who received intact cord resuscitation or cord clamping prior to resuscitation after birth

NEC: necrotizing enterocolitis, DCC: delayed cord clamping, ECC: early cord clamping, RR: risk ratio, 95%-CI: 95% confidence interval, CC: cord clamping

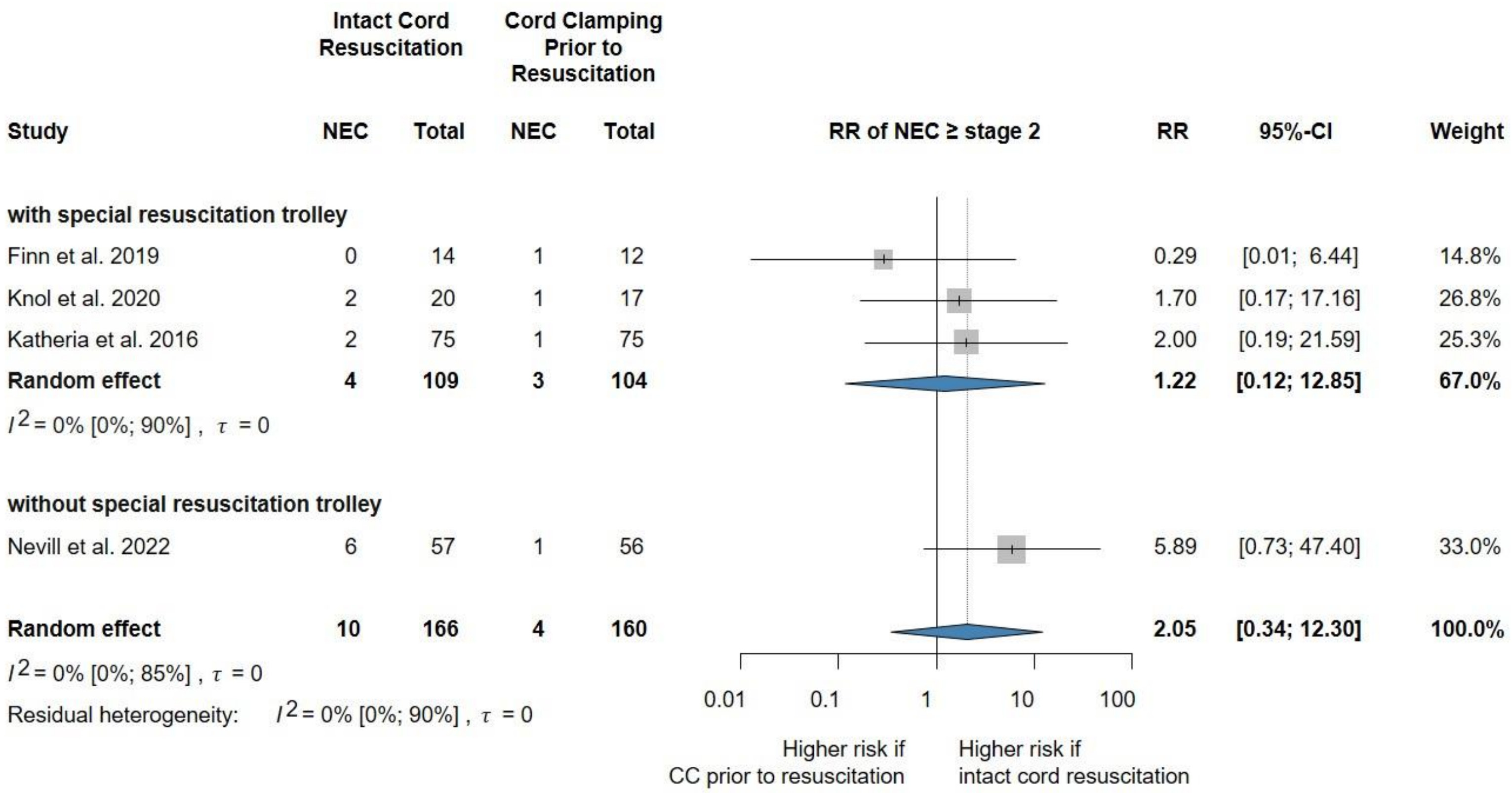

Supplementary Figure 9. Forest plot representing the risk ratio of NEC ≥ stage 2 in infants who received intact cord resuscitation (with or without special resuscitation trolley) or cord clamping prior to resuscitation after birth

NEC: necrotizing enterocolitis, RR: risk ratio, 95%-CI: 95% confidence interval, CC: cord clamping

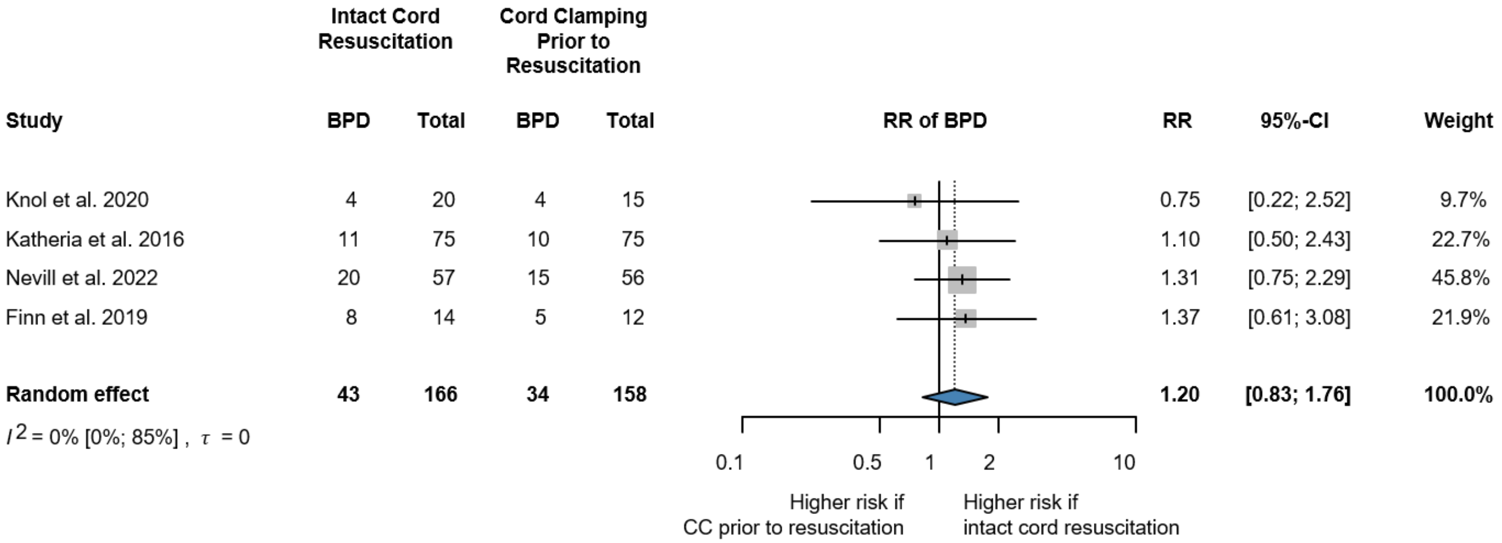

Supplementary Figure 10. Forest plot representing the risk ratio of BPD in infants who received intact cord resuscitation or cord clamping prior to resuscitation after birth

BPD: bronchopulmonary dysplasia, RR: risk ratio, 95%-CI: 95% confidence interval, CC: cord clamping

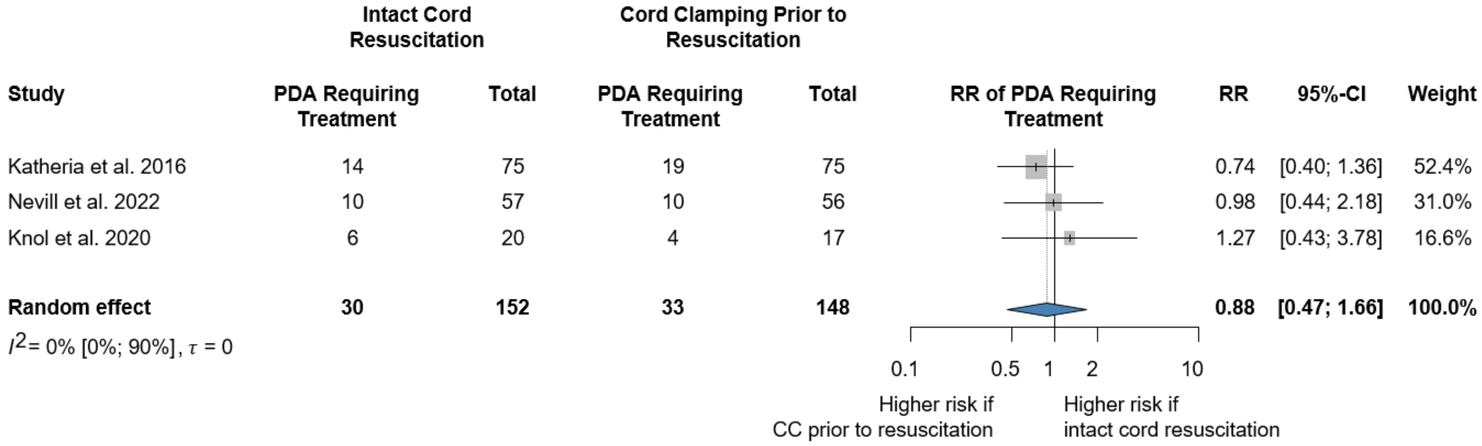

Supplementary Figure 11. Forest plot representing the risk ratio of PDA requiring treatment in infants who received intact cord resuscitation or cord clamping prior to resuscitation after birth

PDA: patent ductus arteriosus, RR: risk ratio, 95%-CI: 95% confidence interval, CC: cord clamping

Study	Intact Cord Resuscitation LOS	Intact Cord Resuscitation Total	Cord Clamping Prior to Resuscitation LOS	Cord Clamping Prior to Resuscitation Total	RR	95%-CI	Weight
Nevill et al. 2022	19	57	22	56	0.85	[0.52; 1.39]	74.6%
Knol et al. 2020	7	20	6	17	0.99	[0.41; 2.39]	23.3%
Finn et al. 2019	2	14	0	12	4.31	[0.23; 81.58]	2.1%
**Random effect**	**28**	**91**	**28**	**85**	**0.91**	**[0.44; 1.87]**	**100.0%**

I^2 = 0% [0%; 90%] , τ = 0

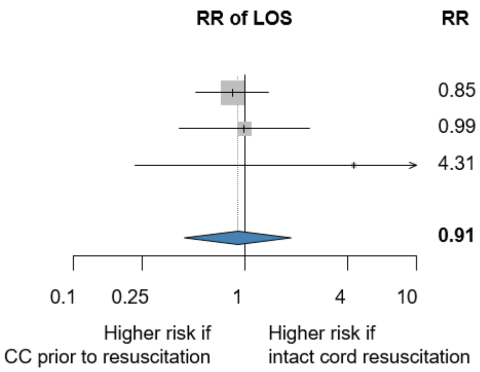

Supplementary Figure 12. Forest plot representing the risk ratio of LOS in infants who received intact cord resuscitation or cord clamping prior to resuscitation after birth

LOS: late-onset neonatal sepsis, RR: risk ratio, 95%-CI: 95% confidence interval, CC: cord clamping

Study	Intact Cord Resuscitation: Need for Transfusion	Intact Cord Resuscitation: Total	Cord Clamping Prior to Resuscitation: Need for Transfusion	Cord Clamping Prior to Resuscitation: Total	RR	95%-CI	Weight
Knol et al. 2020	4	20	6	17	0.57	[0.19; 1.68]	6.3%
Nevill et al. 2022	16	57	17	56	0.92	[0.52; 1.64]	22.6%
Finn et al. 2019	9	14	8	12	0.96	[0.55; 1.69]	23.9%
Katheria et al. 2016	30	75	29	75	1.03	[0.70; 1.54]	47.2%
**Random effect**	**59**	**166**	**60**	**160**	**0.95**	**[0.73; 1.25]**	**100.0%**

$I^2 = 0\%$ [0%; 85%], $\tau = 0$

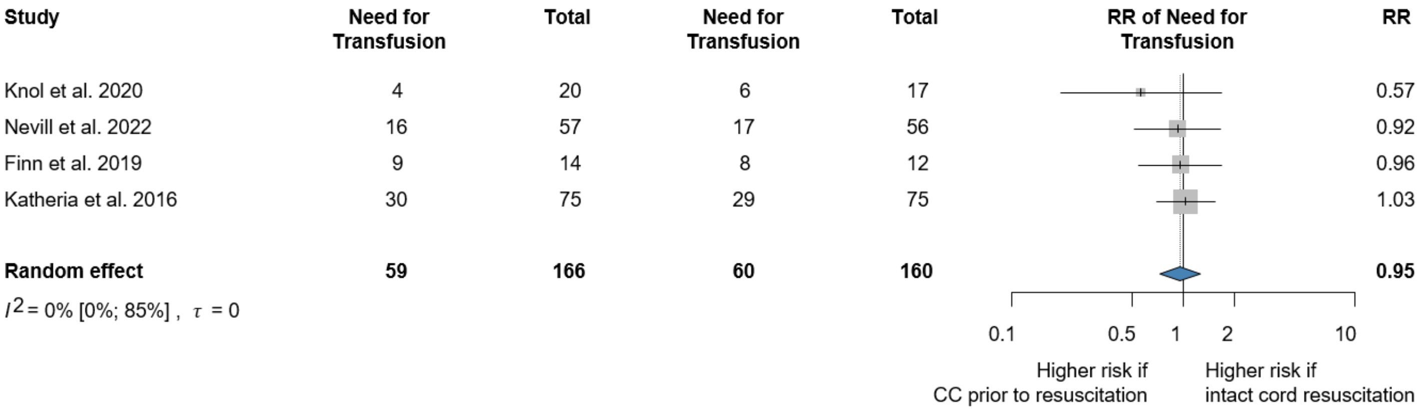

Supplementary Figure 13. Forest plot representing the risk ratio of need for blood transfusion in infants who received intact cord resuscitation or cord clamping prior to resuscitation after birth

RR: risk ratio, 95%-CI: 95% confidence interval, CC: cord clamping

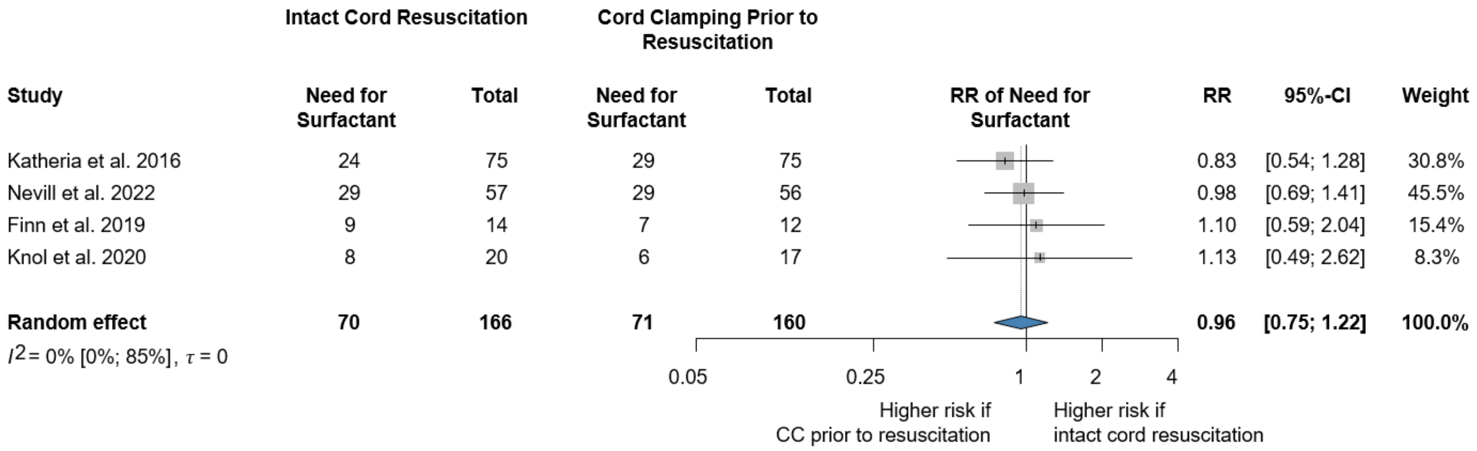

Supplementary Figure 14. Forest plot representing the risk ratio of need for surfactant therapy in infants who received intact cord resuscitation or cord clamping prior to resuscitation after birth

RR: risk ratio, 95%-CI: 95% confidence interval, CC: cord clamping

Study	Intact Cord Resuscitation: Need for Phototherapy	Intact Cord Resuscitation: Total	Cord Clamping Prior to Resuscitation: Need for Phototherapy	Cord Clamping Prior to Resuscitation: Total	RR	95%-CI	Weight
Nevill et al. 2022	43	57	44	56	0.96	[0.78; 1.17]	29.8%
Knol et al. 2020	20	20	15	17	1.13	[0.95; 1.34]	42.7%
Finn et al. 2019	14	14	10	12	1.19	[0.93; 1.52]	20.7%
Raina et al. 2022	28	71	28	91	1.28	[0.84; 1.95]	6.8%
**Random effect**	**105**	**162**	**97**	**176**	**1.10**	**[0.92; 1.30]**	**100.0%**

$I^2 = 0\%$ [0%; 85%], $\tau = 0$

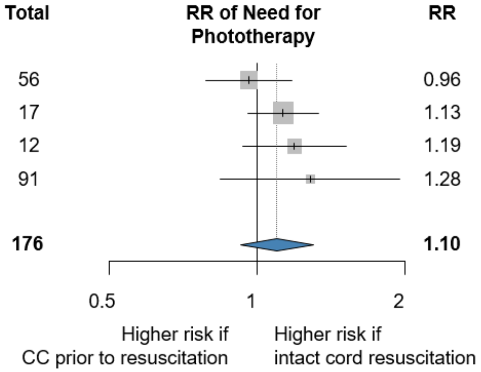

Supplementary Figure 15. Forest plot representing the risk ratio of need for phototherapy in infants who received intact cord resuscitation or cord clamping prior to resuscitation after birth

RR: risk ratio, 95%-CI: 95% confidence interval, CC: cord clamping

Study	Intact Cord Resuscitation: IVH	Intact Cord Resuscitation: Total	Cord Clamping Prior to Resuscitation: IVH	Cord Clamping Prior to Resuscitation: Total	RR	95%-CI	Weight
**with special resuscitation trolley**							
Knol et al. 2020	3	20	2	17	1.27	[0.24; 6.76]	8.1%
Katheria et al. 2016	12	75	8	75	1.50	[0.65; 3.46]	32.3%
**without special resuscitation trolley**							
Nevill et al. 2022	16	57	14	56	1.12	[0.61; 2.08]	59.6%
**Random effect**	**31**	**152**	**24**	**148**	**1.25**	**[0.77; 2.00]**	**100.0%**

$I^2 = 0\%$ [0%; 90%] , $\tau = 0$

Residual heterogeneity: $I^2 = 0\%$, $\tau = 0$

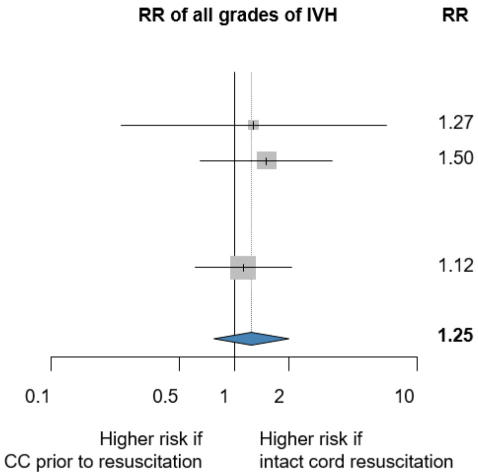

Supplementary Figure 16. Forest plot representing the risk ratio of all grades of IVH in infants who received intact cord resuscitation (with or without special resuscitation trolley) or cord clamping prior to resuscitation after birth

IVH: intraventricular hemorrhage, RR: risk ratio, 95%-CI: 95% confidence interval, CC: cord clamping

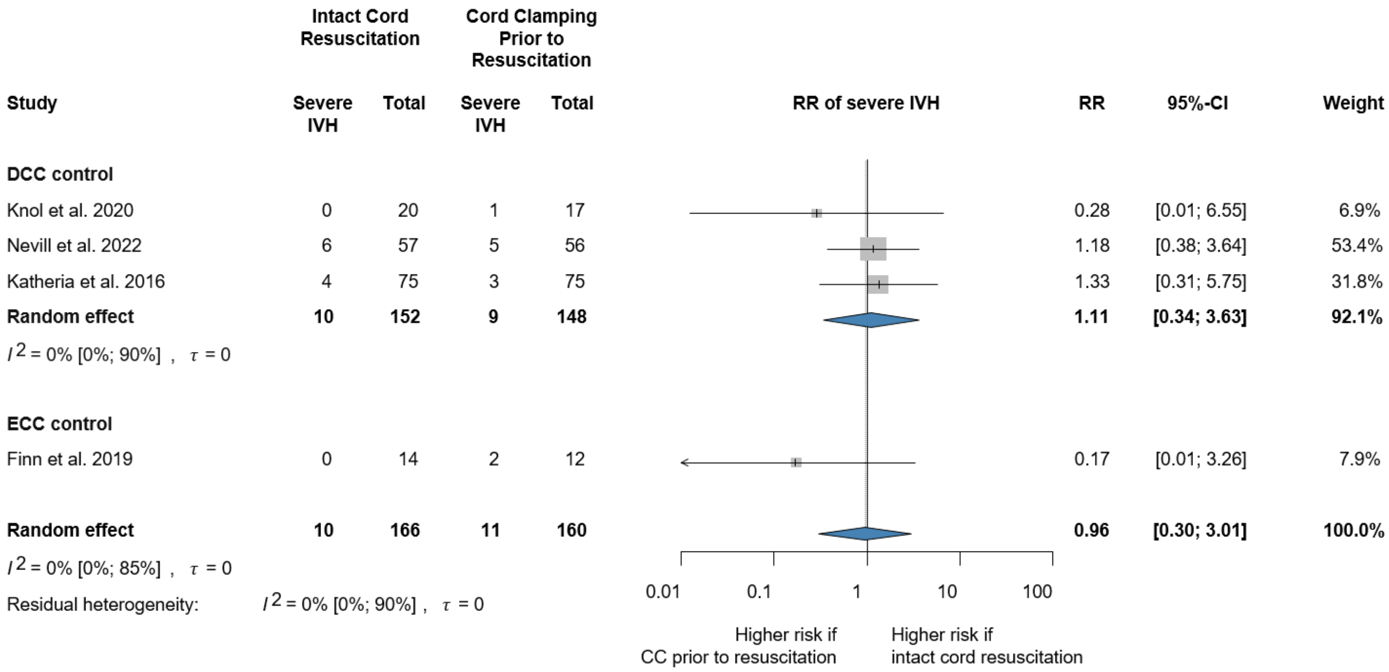

Supplementary Figure 17. Forest plot representing the risk ratio of severe IVH (≥ grade 3) in DCC vs. ECC control subgroups among infants who received intact cord resuscitation or cord clamping prior to resuscitation after birth

IVH: intraventricular hemorrhage, DCC: delayed cord clamping, ECC: early cord clamping, RR: risk ratio, 95%-CI: 95% confidence interval, CC: cord clamping

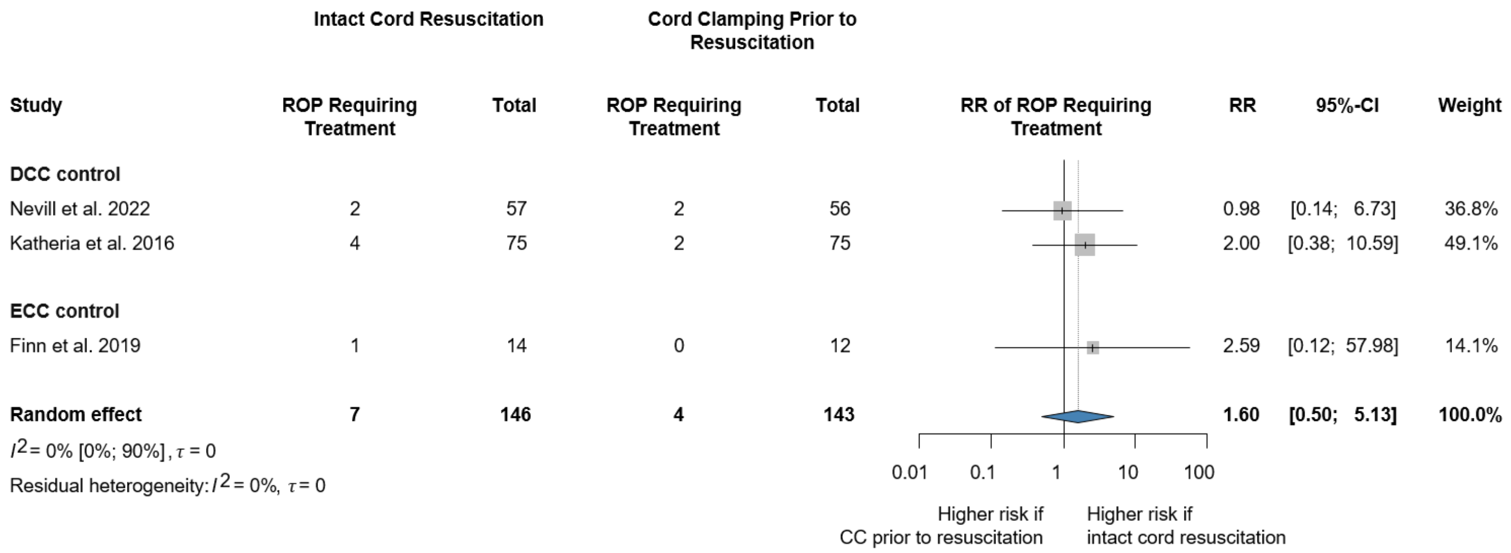

Supplementary Figure 18. Forest plot representing the risk ratio of <u>ROP requiring treatment</u> in DCC vs. ECC control subgroups among infants who received intact cord resuscitation or cord clamping prior to resuscitation after birth

ROP: retinopathy of the prematurity, DCC: delayed cord clamping, ECC: early cord clamping, RR: risk ratio, 95%-CI: 95% confidence interval, CC: cord clamping

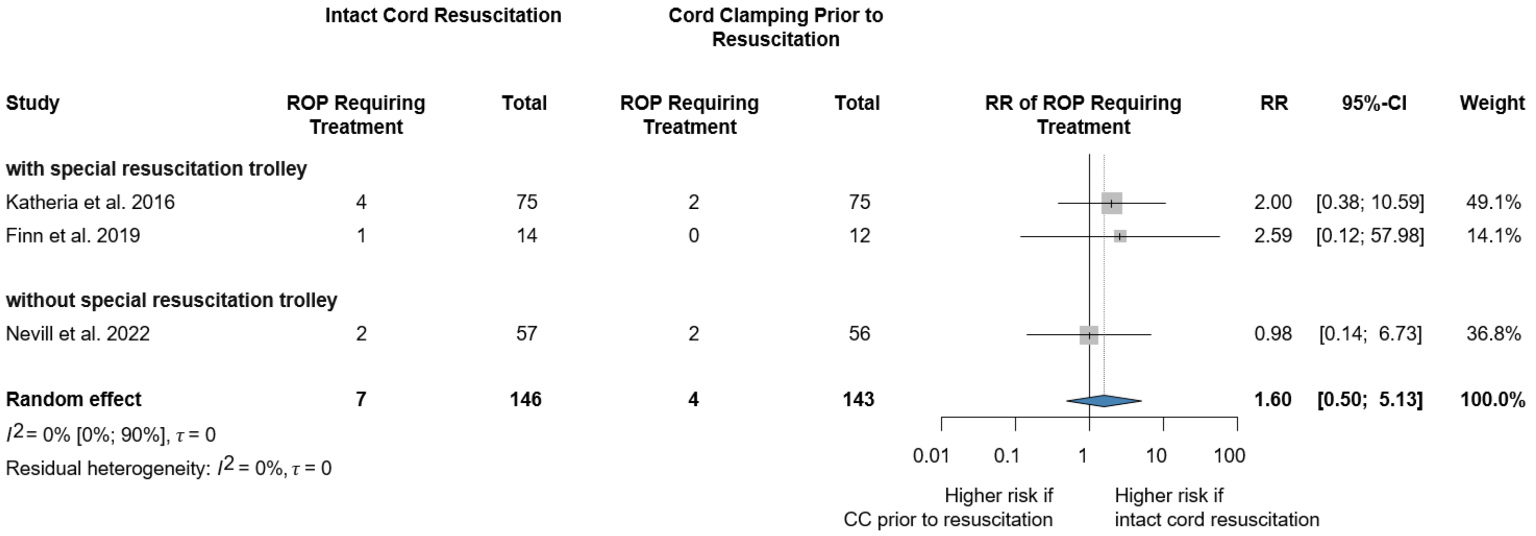

Supplementary Figure 19. Forest plot representing the risk ratio of <u>ROP requiring treatment</u> in infants who received intact cord resuscitation (with or without special resuscitation trolley) or cord clamping prior to resuscitation after birth

ROP: retinopathy of the prematurity, RR: risk ratio, 95%-CI: 95% confidence interval, CC: cord clamping

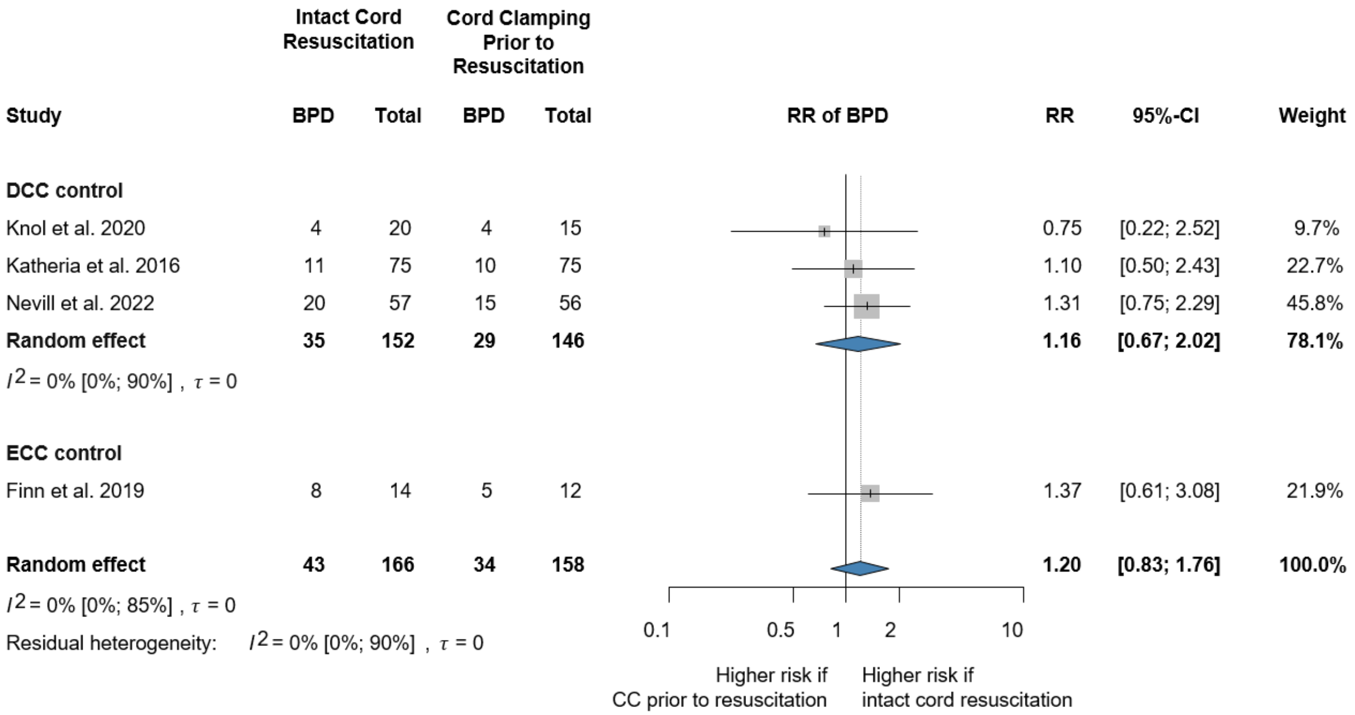

Supplementary Figure 20. Forest plot representing the risk ratio of BPD in DCC vs. ECC control subgroups among infants who received intact cord resuscitation or cord clamping prior to resuscitation after birth

BPD: bronchopulmonary dysplasia, DCC: delayed cord clamping, ECC: early cord clamping, RR: risk ratio, 95%-CI: 95% confidence interval, CC: cord clamping

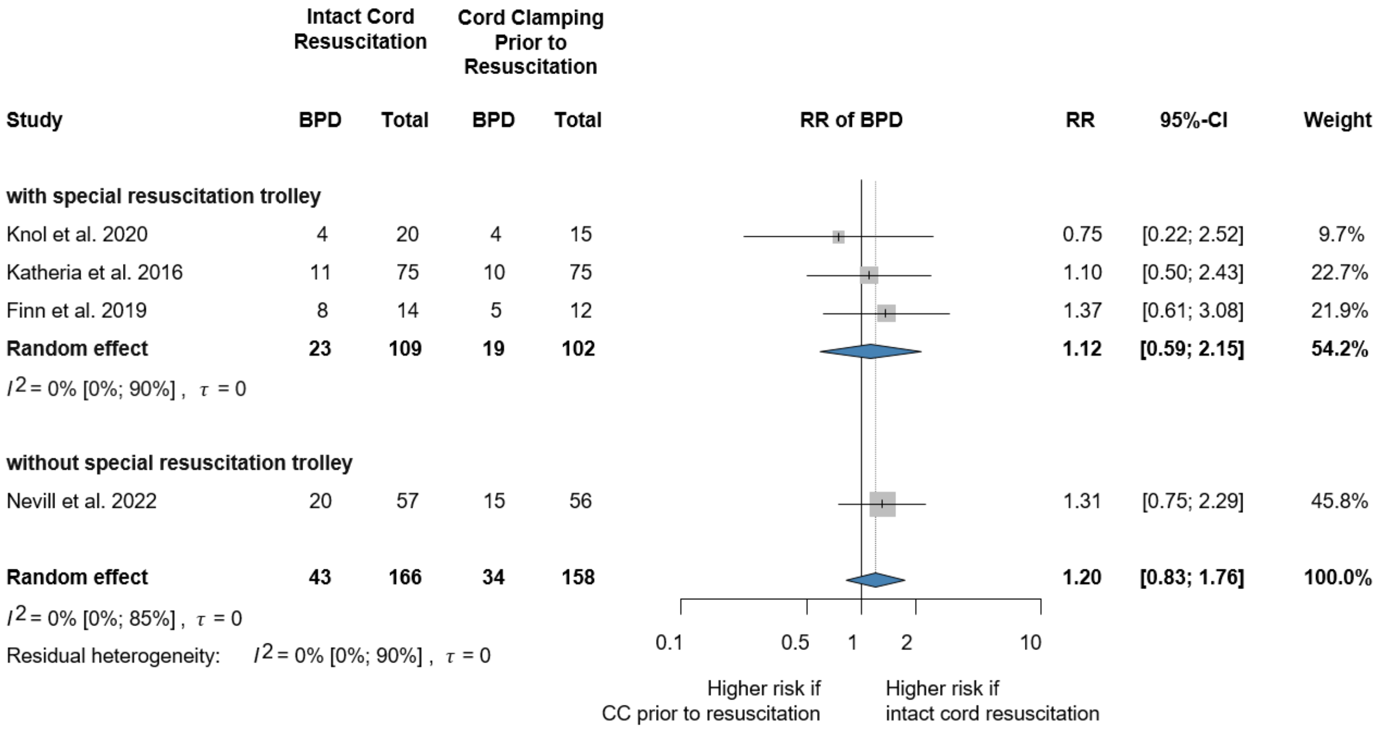

Supplementary Figure 21. Forest plot representing the risk ratio of BPD in infants who received intact cord resuscitation (with or without special resuscitation trolley) or cord clamping prior to resuscitation after birth

BPD: bronchopulmonary dysplasia, RR: risk ratio, 95%-CI: 95% confidence interval, CC: cord clamping

Study	Intact Cord Resuscitation: PDA Requiring Treatment	Intact Cord Resuscitation: Total	Cord Clamping Prior to Resuscitation: PDA Requiring Treatment	Cord Clamping Prior to Resuscitation: Total	RR of PDA Requiring Treatment	RR	95%-CI	Weight
**with special resuscitation trolley**								
Katheria et al. 2016	14	75	19	75		0.74	[0.40; 1.36]	52.4%
Knol et al. 2020	6	20	4	17		1.27	[0.43; 3.78]	16.6%
**without special resuscitation trolley**								
Nevill et al. 2022	10	57	10	56		0.98	[0.44; 2.18]	31.0%
**Random effect**	**30**	**152**	**33**	**148**		**0.88**	**[0.47; 1.66]**	**100.0%**

$I^2 = 0\%$ [0%; 90%], $\tau = 0$

Residual heterogeneity: $I^2 = 0\%, \tau = 0$

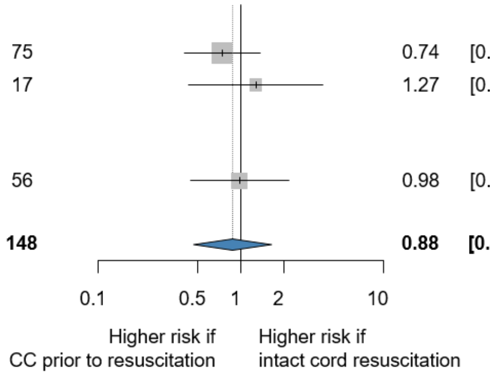

Supplementary Figure 22. Forest plot representing the risk ratio of PDA requiring treatment in infants who received intact cord resuscitation (with or without special resuscitation trolley) or cord clamping prior to resuscitation after birth

PDA: patent ductus arteriosus, RR: risk ratio, 95%-CI: 95% confidence interval, CC: cord clamping

Study	Intact Cord Resuscitation: Need for Transfusion	Intact Cord Resuscitation: Total	Cord Clamping Prior to Resuscitation: Need for Transfusion	Cord Clamping Prior to Resuscitation: Total	RR	95%-CI	Weight
**DCC control**							
Knol et al. 2020	4	20	6	17	0.57	[0.19; 1.68]	6.3%
Nevill et al. 2022	16	57	17	56	0.92	[0.52; 1.64]	22.6%
Katheria et al. 2016	30	75	29	75	1.03	[0.70; 1.54]	47.2%
**Random effect**	**50**	**152**	**52**	**148**	**0.95**	**[0.58; 1.57]**	**76.1%**
I^2 = 0% [0%; 90%], τ = 0							
**ECC control**							
Finn et al. 2019	9	14	8	12	0.96	[0.55; 1.69]	23.9%
**Random effect**	**59**	**166**	**60**	**160**	**0.95**	**[0.73; 1.25]**	**100.0%**
I^2 = 0% [0%; 85%], τ = 0							
Residual heterogeneity: I^2 = 0% [0%; 90%], τ = 0							

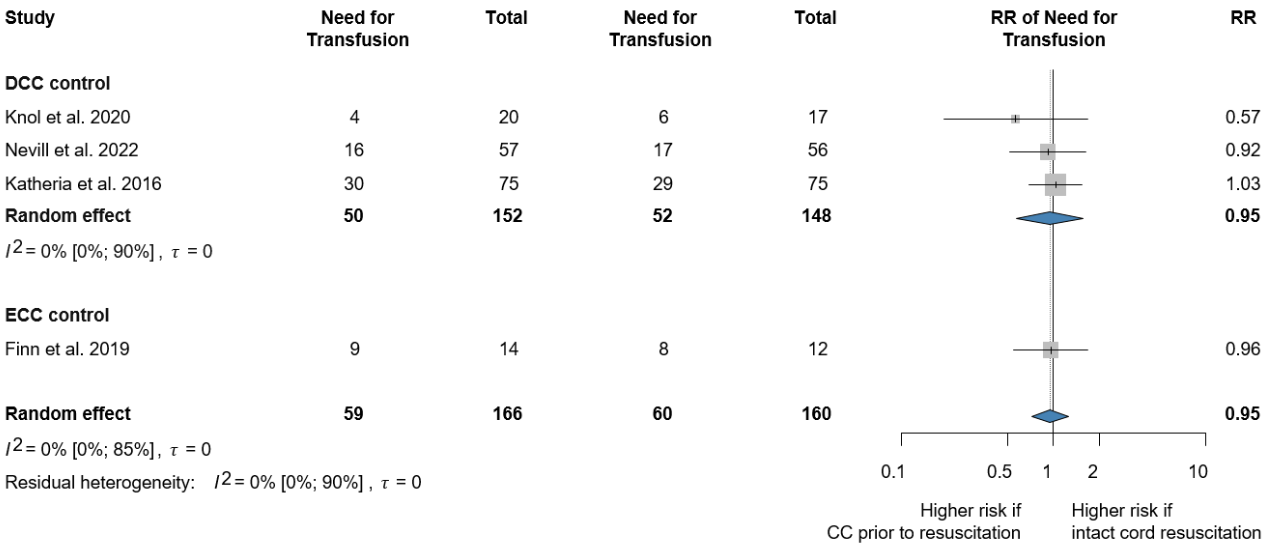

Supplementary Figure 23. Forest plot representing the risk ratio of <u>need for blood transfusion</u> in DCC vs. ECC control subgroups among infants who received intact cord resuscitation or cord clamping prior to resuscitation after birth

DCC: delayed cord clamping, ECC: early cord clamping, RR: risk ratio, 95%-CI: 95% confidence interval, CC: cord clamping

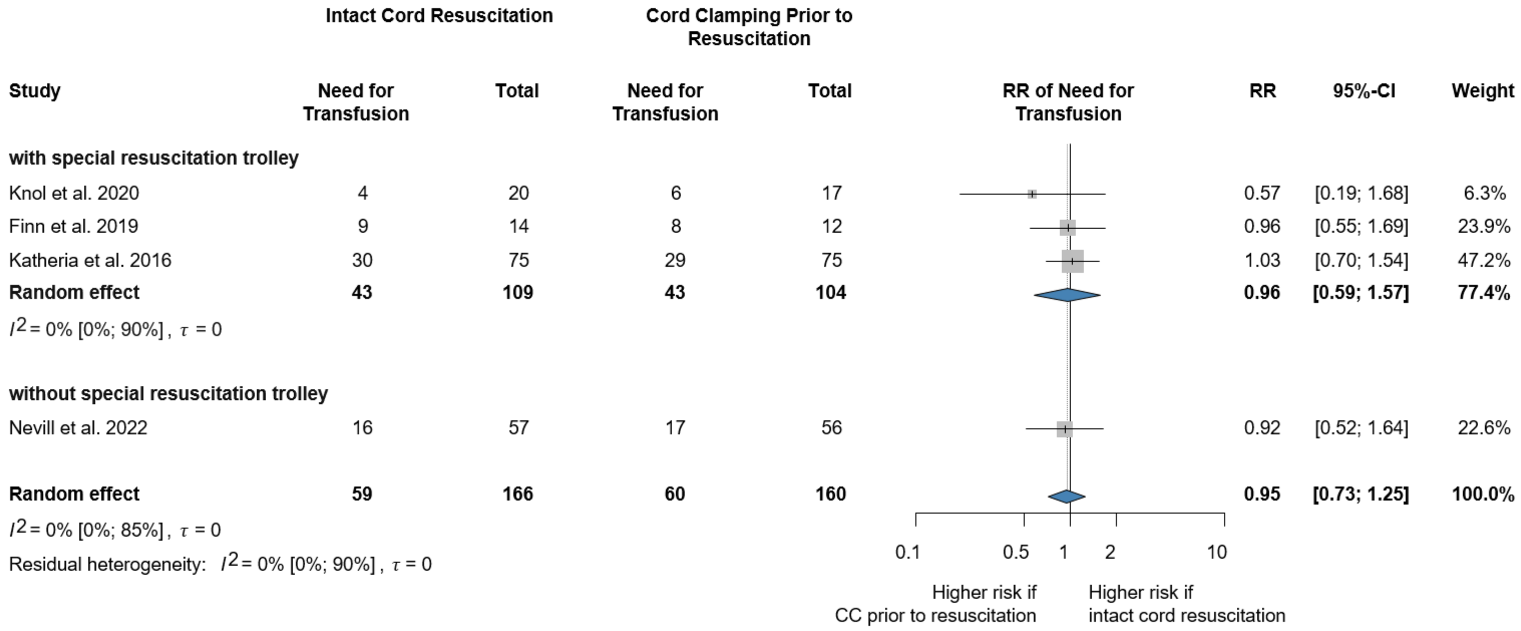

Supplementary Figure 24. Forest plot representing the risk ratio of <u>need for blood transfusion</u> in infants who received intact cord resuscitation (with or without special resuscitation trolley) or cord clamping prior to resuscitation after birth

RR: risk ratio, 95%-CI: 95% confidence interval, CC: cord clamping

Study	Intact Cord Resuscitation: Need for Phototherapy	Intact Cord Resuscitation: Total	Cord Clamping Prior to Resuscitation: Need for Phototherapy	Cord Clamping Prior to Resuscitation: Total	RR	95%-CI	Weight
**DCC control**							
Nevill et al. 2022	43	57	44	56	0.96	[0.78; 1.17]	29.8%
Knol et al. 2020	20	20	15	17	1.13	[0.95; 1.34]	42.7%
**ECC control**							
Finn et al. 2019	14	14	10	12	1.19	[0.93; 1.52]	20.7%
Raina et al. 2022	28	71	28	91	1.28	[0.84; 1.95]	6.8%
**Random effect**	**105**	**162**	**97**	**176**	**1.10**	**[0.92; 1.30]**	**100.0%**

I^2 = 0% [0%; 85%], τ = 0

Residual heterogeneity: I^2 = 0% [0%; 90%], τ = 0

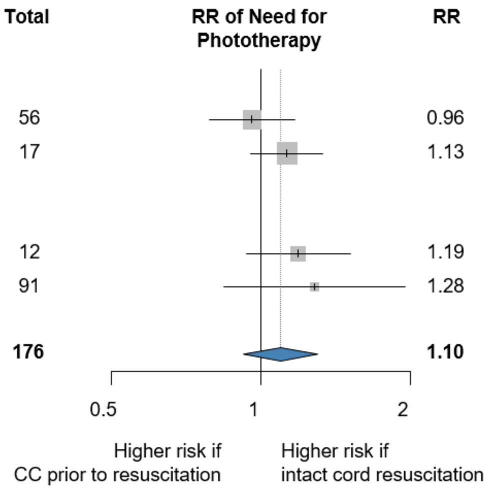

Supplementary Figure 25. Forest plot representing the risk ratio of <u>need for phototherapy</u> in DCC vs. ECC control subgroups among infants who received intact cord resuscitation or cord clamping prior to resuscitation after birth

DCC: delayed cord clamping, ECC: early cord clamping, RR: risk ratio, 95%-CI: 95% confidence interval, CC: cord clamping

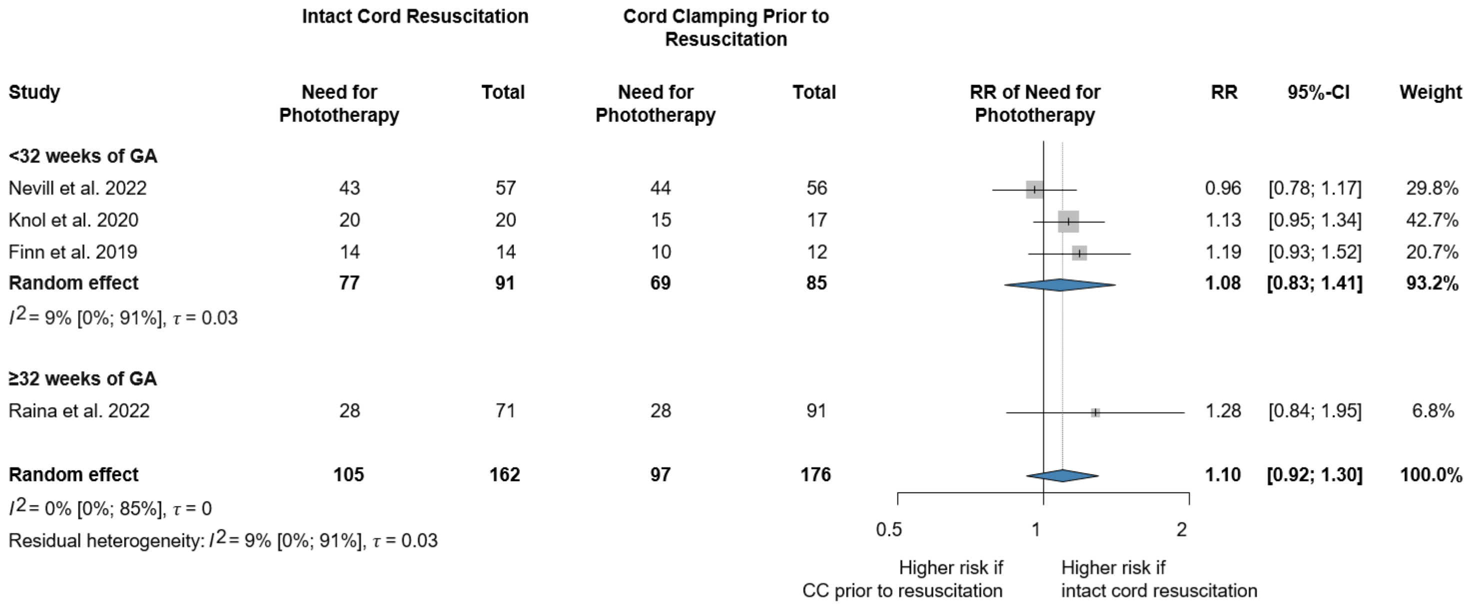

Supplementary Figure 26. Forest plot representing the risk ratio of <u>need for phototherapy</u> in infants (< and ≥32 weeks of GA) who received intact cord resuscitation or cord clamping prior to resuscitation after birth

GA: gestational age, RR: risk ratio, 95%-CI: 95% confidence interval, CC: cord clamping

	Intact Cord Resuscitation		Cord Clamping Prior to Resuscitation				
Study	Need for Phototherapy	Total	Need for Phototherapy	Total	RR	95%-CI	Weight
**with special resuscitation trolley**							
Knol et al. 2020	20	20	15	17	1.13	[0.95; 1.34]	42.7%
Finn et al. 2019	14	14	10	12	1.19	[0.93; 1.52]	20.7%
Raina et al. 2022	28	71	28	91	1.28	[0.84; 1.95]	6.8%
**Random effect**	**62**	**105**	**53**	**120**	**1.16**	**[1.02; 1.32]**	**70.2%**
$I^2 = 0\%$ [0%; 90%], $\tau = 0$							
**without special resuscitation trolley**							
Nevill et al. 2022	43	57	44	56	0.96	[0.78; 1.17]	29.8%
**Random effect**	**105**	**162**	**97**	**176**	**1.10**	**[0.92; 1.30]**	**100.0%**
$I^2 = 0\%$ [0%; 85%], $\tau = 0$							
Residual heterogeneity: $I^2 = 0\%$ [0%; 90%], $\tau = 0$							

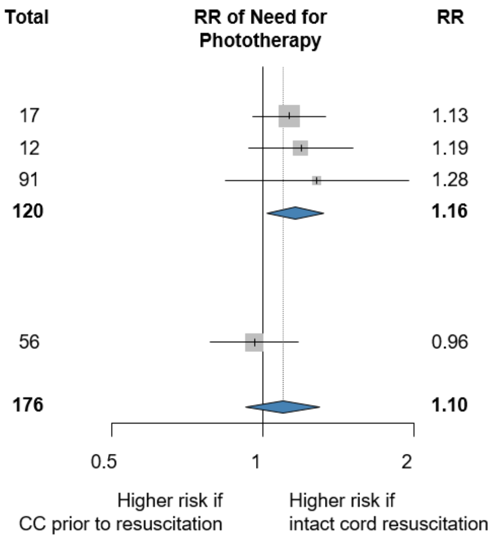

Supplementary Figure 27. Forest plot representing the risk ratio of need for phototherapy in infants who received intact cord resuscitation (with or without special resuscitation trolley) or cord clamping prior to resuscitation after birth

RR: risk ratio, 95%-CI: 95% confidence interval, CC: cord clamping

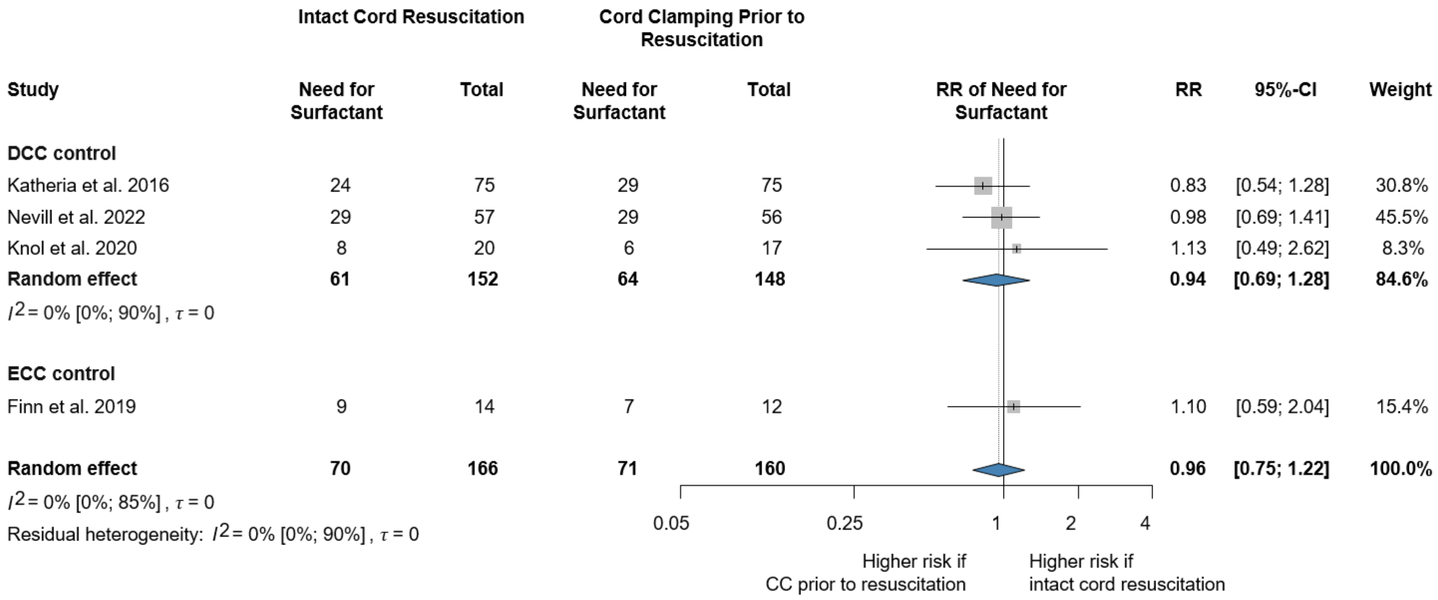

Supplementary Figure 28. Forest plot representing the risk ratio of need for surfactant therapy in DCC vs. ECC control subgroups among infants who received intact cord resuscitation or cord clamping prior to resuscitation after birth

DCC: delayed cord clamping, ECC: early cord clamping, RR: risk ratio, 95%-CI: 95% confidence interval, CC: cord clamping

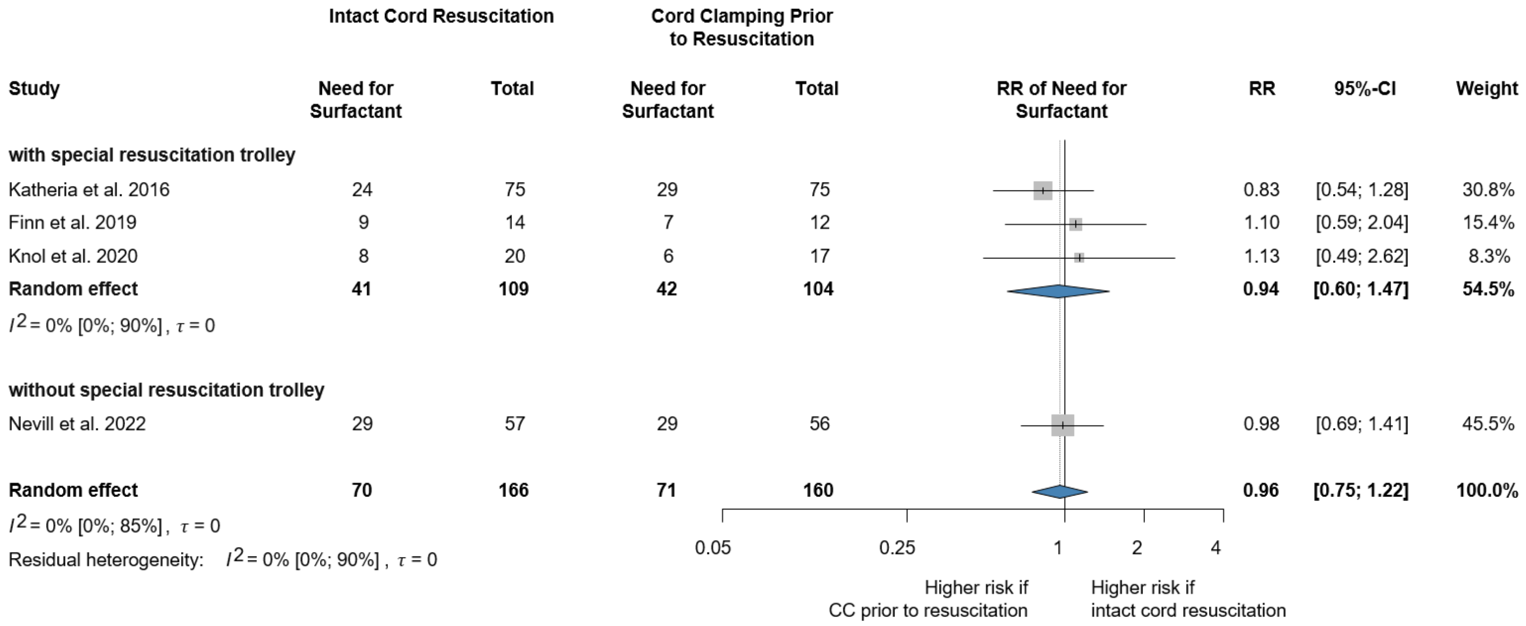

Supplementary Figure 29. Forest plot representing the risk ratio of need for surfactant therapy in infants who received intact cord resuscitation (with or without special resuscitation trolley) or cord clamping prior to resuscitation after birth

RR: risk ratio, 95%-CI: 95% confidence interval, CC: cord clamping

Study	Intact Cord Resuscitation LOS	Intact Cord Resuscitation Total	Cord Clamping Prior to Resuscitation LOS	Cord Clamping Prior to Resuscitation Total	RR	95%-CI	Weight
**DCC control**							
Nevill et al. 2022	19	57	22	56	0.85	[0.52; 1.39]	74.6%
Knol et al. 2020	7	20	6	17	0.99	[0.41; 2.39]	23.3%
**ECC control**							
Finn et al. 2019	2	14	0	12	4.31	[0.23; 81.58]	2.1%
**Random effect**	**28**	**91**	**28**	**85**	**0.91**	**[0.44; 1.87]**	**100.0%**

$I^2 = 0\%$ [0%; 90%], $\tau = 0$

Residual heterogeneity: $I^2 = 0\%$, $\tau = 0$

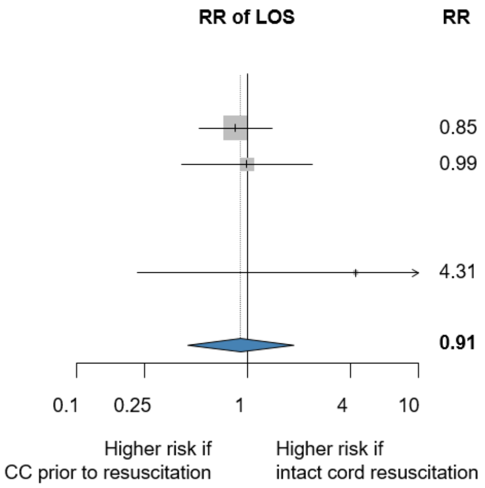

Supplementary Figure 30. Forest plot representing the risk ratio of LOS in DCC vs. ECC control subgroups among infants who received intact cord resuscitation or cord clamping prior to resuscitation after birth

LOS: late-onset neonatal sepsis, DCC: delayed cord clamping, ECC: early cord clamping, RR: risk ratio, 95%-CI: 95% confidence interval, CC: cord clamping

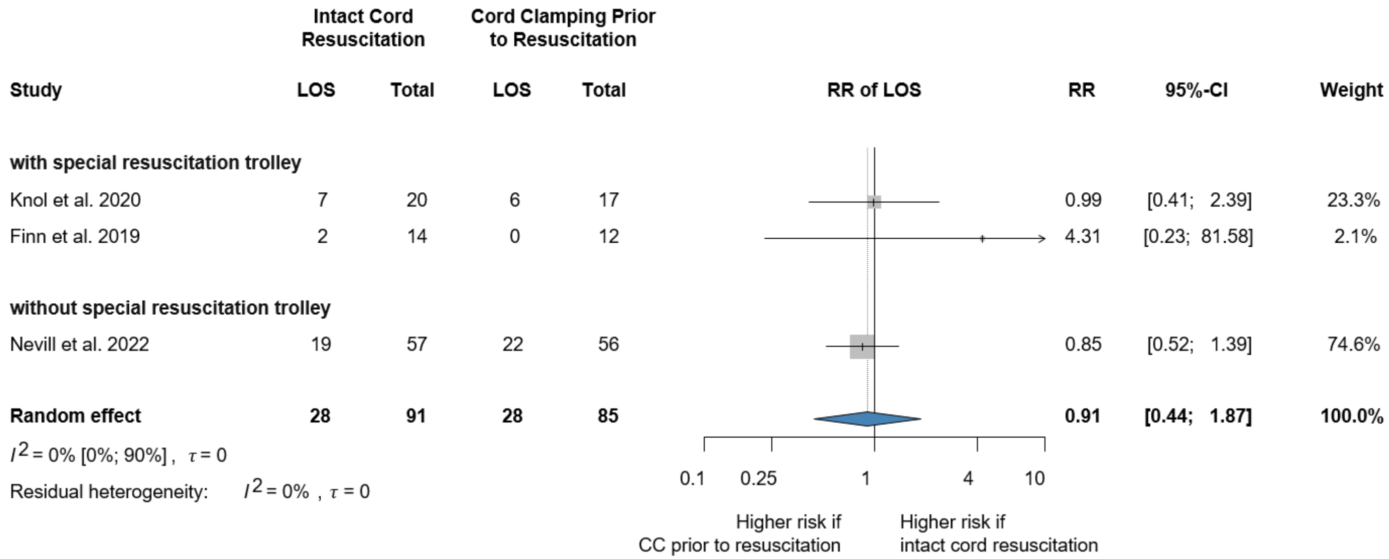

Supplementary Figure 31. Forest plot representing the risk ratio of <u>LOS</u> in infants who received intact cord resuscitation (with or without special resuscitation trolley) or cord clamping prior to resuscitation after birth

LOS: late-onset neonatal sepsis, RR: risk ratio, 95%-CI: 95% confidence interval, CC: cord clamping

Publication bias assessment and influental analyses did not prove publication bias or potential outlier publications (results not shown).

9. Visualization of risk of bias assessment (RoB2)

Risk of bias domains

Study	D1	D2	D3	D4	D5	Overall
Andersson et al. 2019, in-hospital mortality	+	X	+	+	+	X
Katheria et al. 2016, in-hospital mortality	+	X	+	+	+	X
Knol et al. 2020, in-hospital mortality	+	X	+	+	+	X
Nevill et al. 2022, in-hospital mortality	+	-	+	+	+	-
Raina et al. 2022, in-hospital mortality	+	+	+	+	+	+
Katheria et al. 2016, IVH all grades	+	X	+	+	+	X
Knol et al. 2020, IVH all grades	+	X	+	+	+	X
Nevill et al. 2022, IVH all grades	+	-	+	+	+	-
Finn et al. 2019, IVH >= grade 3	+	X	+	+	+	X
Katheria et al. 2016, IVH >= grade 3	+	X	+	+	+	X
Knol et al. 2020, IVH >= grade 3	+	X	+	+	+	X
Nevill et al. 2022, IVH >= grade 3	+	-	+	+	+	-
Finn et al. 2019, BPD	+	X	+	+	+	X
Katheria et al. 2016, BPD	+	X	+	+	+	X
Knol et al. 2020, BPD	+	X	+	+	+	X
Nevill et al. 2022, BPD	+	-	+	+	+	-
Katheria et al. 2016, PDA requiring treatment	+	X	+	+	+	X
Knol et al. 2020, PDA requiring treatment	+	X	+	+	+	X
Nevill et al. 2022, PDA requiring treatment	+	-	+	+	+	-
Finn et al. 2019, NEC >= grade 2	+	X	+	+	+	X
Katheria et al. 2016, NEC >= grade 2	+	X	+	+	+	X
Knol et al. 2020, NEC >= grade 2	+	X	+	+	+	X
Nevill et al. 2022, NEC >= grade 2	+	-	+	+	+	-
Finn et al. 2019, ROP requiring treatment	+	X	+	+	+	X
Katheria et al. 2016, ROP requiring treatment	+	X	+	+	+	X
Nevill et al. 2022, ROP requiring treatment	+	-	+	+	+	-
Finn et al. 2019, need for blood transfusion	+	X	+	+	+	X
Katheria et al. 2016, need for blood transfusion	+	X	+	+	+	X
Knol et al. 2020, need for blood transfusion	+	X	+	+	+	X
Nevill et al. 2022, need for blood transfusion	+	-	+	+	+	-
Finn et al. 2019, need for phototherapy	+	X	+	+	+	X
Knol et al. 2020, need for phototherapy	+	X	+	+	+	X
Nevill et al. 2022, need for phototherapy	+	-	+	+	+	-
Raina et al. 2022, need for phototherapy	+	+	+	+	+	+
Finn et al. 2019, need for surfactant	+	X	+	+	+	X
Katheria et al. 2016, need for surfactant	+	X	+	+	+	X
Knol et al. 2020, need for surfactant	+	X	+	+	+	X
Nevill et al. 2022, need for surfactant	+	-	+	+	+	-
Finn et al. 2019, late onset sepsis	+	X	+	+	+	X
Knol et al. 2020, late onset sepsis	+	X	+	+	+	X
Nevill et al. 2022, late onset sepsis	+	-	+	+	+	-
Andersson et al. 2019, SpO2 at 5 mins	+	X	+	+	+	X
Nevill et al. 2022, SpO2 at 5 mins	+	-	+	+	+	-
Raina et al. 2022, SpO2 at 5 mins	+	+	+	+	+	+
Andersson et al. 2019, SpO2 at 10 mins	+	X	+	+	+	X
Nevill et al. 2022, SpO2 at 10 mins	+	-	+	+	+	-
Raina et al. 2022, SpO2 at 10 mins	+	+	+	+	+	+
Andersson et al. 2019, APGAR score at 1 min	+	X	+	-	+	X
Finn et al. 2019, APGAR score at 1 min	+	X	+	-	+	X
Katheria et al. 2016, APGAR score at 1 min	+	X	+	-	+	X
Knol et al. 2020, APGAR score at 1 min	+	X	+	-	+	X
Nevill et al. 2022, APGAR score at 1 min	+	-	+	-	+	-
Raina et al. 2022, APGAR score at 1 min	+	+	+	-	+	-
Andersson et al. 2019, APGAR score at 5 mins	+	X	+	-	+	X
Katheria et al. 2016, APGAR score at 5 mins	+	X	+	+	+	X
Knol et al. 2020, APGAR score at 5 mins	+	X	+	-	+	X
Nevill et al. 2022, APGAR score at 5 mins	+	-	+	-	+	-
Raina et al. 2022, APGAR score at 5 mins	+	+	+	-	+	-
Andersson et al. 2019, APGAR score at 10 mins	+	X	+	-	+	X
Knol et al. 2020, APGAR score at 10 mins	+	X	+	-	+	X
Raina et al. 2022, APGAR score at 10 mins	+	+	+	-	+	-
Finn et al. 2019, temperature at admission to NICU	+	X	+	+	+	X
Katheria et al. 2016, temperature at admission to NICU	+	X	+	+	+	X
Knol et al. 2020, temperature at admission to NICU	+	X	+	+	+	X

Domains:
D1: Bias arising from the randomization process.
D2: Bias due to deviations from intended intervention.
D3: Bias due to missing outcome data.
D4: Bias in measurement of the outcome.
D5: Bias in selection of the reported result.

Judgement
X High
- Some concerns
+ Low

Supplementary Table 4. Overall risk of bias assessment of the included studies with each outcomes comparing intact cord resuscitation to cord clamping prior to resuscitation

IVH: intraventricular hemorrhage, BPD: bronchopulmonary dysplasia, PDA: patent ductus arteriosus, NEC: necrotizing enterocolitis, ROP: retinopathy of the prematurity, LOS: late-onset neonatal sepsis, SpO_2: oxygen saturation level by pulse oximetry, NICU: neonatal intensive unit care

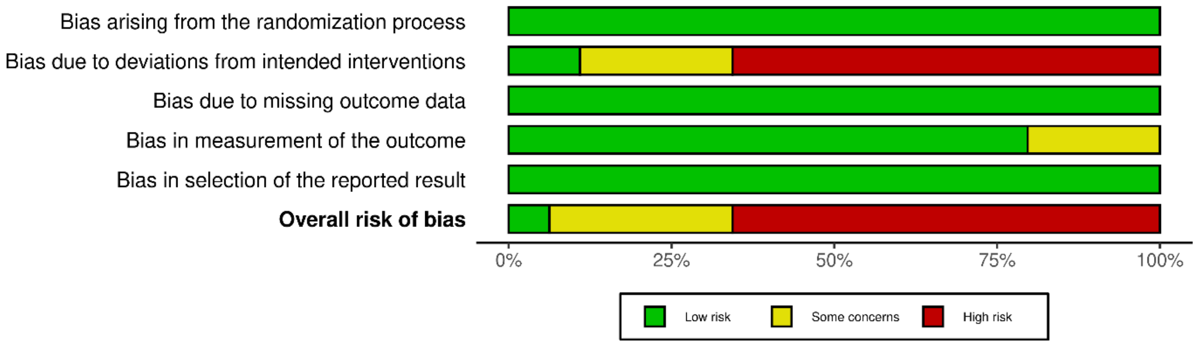

Supplementary Table 5. Overall risk of bias assessment by domains of the included studies with each outcomes comparing intact cord resuscitation to cord clamping prior to resuscitation

10. GRADE assessment (level of evidence)

Question: Intact cord resuscitation compared to cord clamping prior to resuscitation for infants who require resuscitation after birth

Certainty assessment							№ of patients		Effect		Certainty	Importance
**№ of studies**	**Study design**	**Risk of bias**	**Inconsistency**	**Indirectness**	**Imprecision**	**Other considerations**	**intact cord resuscitation**	**cord clamping prior to resuscitation**	**Relative (95% CI)**	**Absolute (95% CI)**		
**In-hospital mortality (assessed with: risk ratio)**												
5	randomised trials	serious	not serious	not serious	serious	none	11/297 (3.7%)	13/287 (4.5%)	**RR 0.89** (0.24 to 3.36)	**5 fewer per 1 000** (from 34 fewer to 107 more)	⊕⊕◯◯ Low	CRITICAL

<table>
<tr><th colspan="7">Certainty assessment</th><th colspan="2">№ of patients</th><th colspan="2">Effect</th><th rowspan="2">Certainty</th><th rowspan="2">Importance</th></tr>
<tr><th>№ of studies</th><th>Study design</th><th>Risk of bias</th><th>Inconsistency</th><th>Indirectness</th><th>Imprecision</th><th>Other considerations</th><th>intact cord resuscitation</th><th>cord clamping prior to resuscitation</th><th>Relative (95% CI)</th><th>Absolute (95% CI)</th></tr>
<tr><td colspan="13">Intraventricular hemorrhage all grades (assessed with: risk ratio)</td></tr>
<tr><td>3</td><td>randomised trials</td><td>serious</td><td>not serious</td><td>not serious</td><td>serious</td><td>none</td><td>31/152 (20.4%)</td><td>24/148 (16.2%)</td><td>RR 1.25 (0.77 to 2.00)</td><td>41 more per 1 000 (from 37 fewer to 162 more)</td><td>⊕⊕◯◯ Low</td><td>CRITICAL</td></tr>
<tr><td colspan="13">Intraventricular hemorrhage >= grade 3 (assessed with: risk ratio)</td></tr>
<tr><td>4</td><td>randomised trials</td><td>serious</td><td>not serious</td><td>not serious</td><td>serious</td><td>none</td><td>10/166 (6.0%)</td><td>11/160 (6.9%)</td><td>RR 0.96 (0.30 to 3.01)</td><td>3 fewer per 1 000 (from 48 fewer to 138 more)</td><td>⊕⊕◯◯ Low</td><td>CRITICAL</td></tr>
</table>

Certainty assessment							№ of patients		Effect		Certainty	Importance
№ of studies	Study design	Risk of bias	Inconsistency	Indirectness	Imprecision	Other considerations	intact cord resuscitation	cord clamping prior to resuscitation	Relative (95% CI)	Absolute (95% CI)		
**Bronchopulmonary dysplasia (assessed with: risk ratio)**												
4	randomised trials	serious	not serious	not serious	serious	none	43/166 (25.9%)	34/158 (21.5%)	**RR 1.20** (0.83 to 1.76)	**43 more per 1 000** (from 37 fewer to 164 more)	⊕⊕◯◯ Low	IMPORTANT
**Patent ductus arteriosus requiring treatment (assessed with: risk ratio)**												
3	randomised trials	serious	not serious	not serious	serious	none	30/152 (19.7%)	33/148 (22.3%)	**RR 0.88** (0.47 to 1.66)	**27 fewer per 1 000** (from 118 fewer to 147 more)	⊕⊕◯◯ Low	IMPORTANT

Certainty assessment							№ of patients		Effect		Certainty	Importance
**№ of studies**	**Study design**	**Risk of bias**	**Inconsistency**	**Indirectness**	**Imprecision**	**Other considerations**	**intact cord resuscitation**	**cord clamping prior to resuscitation**	**Relative (95% CI)**	**Absolute (95% CI)**		
**Necrotizing enterocolitis >= grade 2 (assessed with: risk ratio)**												
4	randomised trials	serious	not serious	not serious	very serious	strong association	10/166 (6.0%)	4/160 (2.5%)	**RR 2.05** (0.34 to 12.30)	**26 more per 1 000** (from 16 fewer to 283 more)	⨁⨁◯◯ Low	CRITICAL
**Retinopathy of Prematurity requiring treatment (assessed with: risk ratio)**												
3	randomised trials	serious	not serious	not serious	very serious	none	7/146 (4.8%)	4/143 (2.8%)	**RR 1.60** (0.50 to 5.13)	**17 more per 1 000** (from 14 fewer to 116 more)	⨁◯◯◯ Very low	IMPORTANT

Certainty assessment							№ of patients		Effect		Certainty	Importance
№ of studies	Study design	Risk of bias	Inconsistency	Indirectness	Imprecision	Other considerations	intact cord resuscitation	cord clamping prior to resuscitation	Relative (95% CI)	Absolute (95% CI)		
**Need for blood transfusion (assessed with: risk ratio)**												
4	randomised trials	serious	not serious	not serious	serious	none	59/166 (35.5%)	60/160 (37.5%)	**RR 0.95** (0.73 to 1.25)	**19 fewer per 1 000** (from 101 fewer to 94 more)	⊕⊕◯◯ Low	IMPORTANT
**Need for phototherapy (assessed with: risk ratio)**												
4	randomised trials	serious	not serious	not serious	serious	none	105/162 (64.8%)	97/176 (55.1%)	**RR 1.10** (0.92 to 1.30)	**55 more per 1 000** (from 44 fewer to 165 more)	⊕⊕◯◯ Low	IMPORTANT

Certainty assessment							№ of patients		Effect		Certainty	Importance
№ of studies	Study design	Risk of bias	Inconsistency	Indirectness	Imprecision	Other considerations	intact cord resuscitation	cord clamping prior to resuscitation	Relative (95% CI)	Absolute (95% CI)		
**Need for surfactant (assessed with: risk ratio)**												
4	randomised trials	serious	not serious	not serious	serious	none	70/166 (42.2%)	71/160 (44.4%)	**RR 0.96** (0.75 to 1.22)	**18 fewer per 1 000** (from 111 fewer to 98 more)	⊕⊕◯◯ Low	IMPORTANT
**Late onset neonatal sepsis (assessed with: risk ratio)**												
3	randomised trials	serious	not serious	not serious	serious	none	28/91 (30.8%)	28/85 (32.9%)	**RR 0.91** (0.44 to 1.87)	**30 fewer per 1 000** (from 184 fewer to 287 more)	⊕⊕◯◯ Low	IMPORTANT

Certainty assessment							№ of patients		Effect		Certainty	Importance
**№ of studies**	**Study design**	**Risk of bias**	**Inconsistency**	**Indirectness**	**Imprecision**	**Other considerations**	**intact cord resuscitation**	**cord clamping prior to resuscitation**	**Relative (95% CI)**	**Absolute (95% CI)**		
**Oxygen saturation level at 5 minutes after birth (assessed with: mean difference; Scale from: 0 to 100)**												
3	randomised trials	serious	not serious	not serious	very serious	none	200	192	-	MD **6.67 % higher** (1.16 lower to 14.5 higher)	⊕◯◯◯ Very low	CRITICAL
**Oxygen saturation level at 10 minutes after birth (assessed with: mean difference; Scale from: 0 to 100)**												
3	randomised trials	serious	not serious	not serious	very serious	none	200	192	-	MD **2.87 % higher** (5.53 lower to 11.28 higher)	⊕◯◯◯ Very low	CRITICAL

Certainty assessment							№ of patients		Effect		Certainty	Importance
**№ of studies**	**Study design**	**Risk of bias**	**Inconsistency**	**Indirectness**	**Imprecision**	**Other considerations**	**intact cord resuscitation**	**cord clamping prior to resuscitation**	**Relative (95% CI)**	**Absolute (95% CI)**		
**APGAR score at 1 minute after birth (assessed with: median difference; Scale from: 0 to 10)**												
6	randomised trials	serious	not serious	not serious	serious	none	299	286	-	MD **0.09 lower** (0.55 lower to 0.36 higher)	⨁⨁◯◯ Low	IMPORTANT
**APGAR score at 5 minutes after birth (assessed with: median difference; Scale from: 0 to 10)**												
5	randomised trials	serious	not serious	not serious	serious	none	211	226	-	MedD **0.03 lower** (0.36 lower to 0.29 higher)	⨁⨁◯◯ Low	IMPORTANT

Certainty assessment							№ of patients		Effect		Certainty	Importance
№ of studies	Study design	Risk of bias	Inconsistency	Indirectness	Imprecision	Other considerations	intact cord resuscitation	cord clamping prior to resuscitation	Relative (95% CI)	Absolute (95% CI)		
**Temperature at admission to NICU (assessed with: mean difference)**												
3	randomised trials	serious	not serious	not serious	serious	none	109	104	-	MD **0.04 Celsius lower** (0.2 lower to 0.12 higher)	⊕⊕◯◯ Low	CRITICAL

Supplementary Table 6. Overall GRADE assessment of our examined outcomes (level of evidence)

N°: numbero, CI: confidence interval; MD: mean difference; RR: risk ratio, MedD: median difference, NICU: neonatal intensive care unit

REFERENCES

1. Luo, D., Wan, X., Liu, J. & Tong, T. Optimally estimating the sample mean from the sample size, median, mid-range, and/or mid-quartile range. *Stat. Methods Med. Res.* **27**, 1785–1805 (2018).
2. Shi, J. *et al.* Optimally estimating the sample standard deviation from the five-number summary. *Res. Synth. Methods* **11**, 641–654 (2020).
3. McGrath, S., Sohn, H., Steele, R. & Benedetti, A. Meta-analysis of the difference of medians. *Biom. J.* **62**, 69–98 (2020).
4. Mantel, N. & Haenszel, W. Statistical Aspects of the Analysis of Data From Retrospective Studies of Disease. *JNCI J. Natl. Cancer Inst.* **22**, 719–748 (1959).
5. Robins, James, Sander Greenland, and Norman E. Breslow. 1986. "A General Estimator for the Variance of the Mantel-Haenszel Odds Ratio." American Journal of Epidemiology, 719–23. https://academic.oup.com/aje/article-abstract/124/5/719/121086?redirectedFrom=fulltext&login=false.
6. Cooper, Harris M., Larry V. Hedges, and Jeff C. Valentine, eds. 2009. The Handbook of Research Synthesis and Meta-Analysis. 2nd ed. New York: Russell Sage Foundation. https://www.russellsage.org/publications/handbook-research-synthesis-and-meta-analysis-second-edition.
7. J. Sweeting, Michael, Alexander J. Sutton, and Paul C. Lambert. 2004. "What to Add to Nothing? Use and Avoidance of Continuity Corrections in Meta-Analysis of Sparse Data." Statistics in Medicine 23 (9): 1351–75. https://doi.org/10.1002/sim.1761. https://onlinelibrary.wiley.com/doi/10.1002/sim.1761.
8. Knapp, G. & Hartung, J. Improved tests for a random effects meta-regression with a single covariate. *Stat. Med.* **22**, 2693–2710 (2003).

9. IntHout, J., Ioannidis, J. P. & Borm, G. F. The Hartung-Knapp-Sidik-Jonkman method for random effects meta-analysis is straightforward and considerably outperforms the standard DerSimonian-Laird method. *BMC Med. Res. Methodol.* **14**, 25 (2014).

10. Jackson, Dan, Martin Law, Gerta Rücker, and Guido Schwarzer. 2017. "The Hartung-Knapp Modification for Random-effects Meta-analysis: A Useful Refinement but Are There Any Residual Concerns?" Statistics in Medicine 36 (25): 3923–34. https://doi.org/10.1002/sim.7411. https://onlinelibrary.wiley.com/doi/10.1002/sim.7411.

11. Paule, R. & Mandel, J. Consensus Values, Regressions, and Weighting Factors. *J. Res. Natl. Bur. Stand.* **94**, 197 (1989).

12. Veroniki, A. A. *et al.* Methods to estimate the between-study variance and its uncertainty in meta-analysis. *Res. Synth. Methods* **7**, 55–79 (2016).

13. Borenstein, Michael, Larry V. Hedges, Julian P. T. Higgins, and Hannah R. Rothstein. 2009. Introduction to Meta-Analysis. Chichester, UK: John Wiley & Sons, Ltd. https://doi.org/10.1002/9780470743386. in (John Wiley & Sons, Ltd, 2009). doi:10.1002/9780470743386.fmatter.

14. Badurdeen, S. *et al.* Physiologically based cord clamping for infants ≥32+0 weeks gestation: A randomised clinical trial and reference percentiles for heart rate and oxygen saturation for infants ≥35+0 weeks gestation. *PLoS Med.* **19**, e1004029 (2022).

15. Deng, R. *et al.* With or Without Nasal Continuous Positive Airway Pressure During Delayed Cord Clamping in Premature Infants <32 Weeks: A Randomized Controlled Trial Using an Intention-To-Treat Analysis. *Front. Pediatr.* **10**, 843372 (2022).

16. Duley, L. *et al.* Randomised trial of cord clamping and initial stabilisation at very preterm birth. *Arch. Dis. Child. Fetal Neonatal Ed.* **103**, F6–F14 (2018).

17. Katheria, A. C. *et al.* Delayed Cord Clamping in Newborns Born at Term at Risk for Resuscitation: A Feasibility Randomized Clinical Trial. *J. Pediatr.* **187**, 313-317.e1 (2017).

18. Hocq, C. *et al.* Hocq, C., Van Grambezen, A., Carkeek, K. et al. Implementing intact cord resuscitation in very preterm infants: feasibility and pitfalls. Eur J Pediatr 182, 1105–1113 (2023). https://doi.org/10.1007/s00431-022-04776-2. https://link.springer.com/article/10.1007/s00431-022-04776-2.

19. Hoeller, N. *et al.* Physiological-based cord clamping stabilised cardiorespiratory parameters in very low birth weight infants. *Acta Paediatr.* **n/a**,.

20. Andersson, O. *et al.* Intact cord resuscitation versus early cord clamping in the treatment of depressed newborn infants during the first 10 minutes of birth (Nepcord III) – a randomized clinical trial. *Matern. Health Neonatol. Perinatol.* **5**, 15 (2019).

21. Knol, R. *et al.* Physiological-based cord clamping in very preterm infants — Randomised controlled trial on effectiveness of stabilisation. *Resuscitation* **147**, 26–33 (2020).

22. Nevill, E., Mildenhall, L. F. J. & Meyer, M. P. Effect of Breathing Support in Very Preterm Infants Not Breathing During Deferred Cord Clamping: A Randomized Controlled Trial (The ABC Study). *J. Pediatr.* **253**, 94-100.e1 (2023).

23. Raina, J. S. *et al.* Resuscitation with Intact Cord Versus Clamped Cord in Late Preterm and Term Neonates: A Randomized Controlled Trial. *J. Pediatr.* **254**, 54-60.e4 (2023).
